# Dynamic Zebrafish Interactome Reveals Transcriptional Mechanisms of Dioxin Toxicity

**DOI:** 10.1371/journal.pone.0010465

**Published:** 2010-05-05

**Authors:** Andrey Alexeyenko, Deena M. Wassenberg, Edward K. Lobenhofer, Jerry Yen, Elwood Linney, Erik L. L. Sonnhammer, Joel N. Meyer

**Affiliations:** 1 Stockholm Bioinformatics Centre, Stockholm University, Stockholm, Sweden; 2 Department of Molecular Genetics and Microbiology, Duke University Medical Center, Durham, North Carolina, United States of America; 3 Cogenics, Morrisville, North Carolina, United States of America; 4 Nicholas School of the Environment, Duke University, Durham, North Carolina, United States of America; Northeastern University, United States of America

## Abstract

**Background:**

In order to generate hypotheses regarding the mechanisms by which 2,3,7,8-tetrachlorodibenzo-*p*-dioxin (dioxin) causes toxicity, we analyzed global gene expression changes in developing zebrafish embryos exposed to this potent toxicant in the context of a dynamic gene network. For this purpose, we also computationally inferred a zebrafish (*Danio rerio*) interactome based on orthologs and interaction data from other eukaryotes.

**Methodology/Principal Findings:**

Using novel computational tools to analyze this interactome, we distinguished between dioxin-dependent and dioxin-independent interactions between proteins, and tracked the temporal propagation of dioxin-dependent transcriptional changes from a few genes that were altered initially, to large groups of biologically coherent genes at later times. The most notable processes altered at later developmental stages were calcium and iron metabolism, embryonic morphogenesis including neuronal and retinal development, a variety of mitochondria-related functions, and generalized stress response (not including induction of antioxidant genes). Within the interactome, many of these responses were connected to cytochrome P4501A (*cyp1a*) as well as other genes that were dioxin-regulated one day after exposure. This suggests that *cyp1a* may play a key role initiating the toxic dysregulation of those processes, rather than serving simply as a passive marker of dioxin exposure, as suggested by earlier research.

**Conclusions/Significance:**

Thus, a powerful microarray experiment coupled with a flexible interactome and multi-pronged interactome tools (which are now made publicly available for microarray analysis and related work) suggest the hypothesis that dioxin, best known in fish as a potent cardioteratogen, has many other targets. Many of these types of toxicity have been observed in mammalian species and are potentially caused by alterations to *cyp1a*.

## Introduction

2,3,7,8-tetrachlorodibenzo-*p*-dioxin (TCDD or dioxin) is a heavily-studied yet poorly-understood pollutant. Observations pertaining to dioxin exposure on humans and other vertebrate species include endocrine disruption, immune system dysfunction, carcinogenesis, and teratogenicity (particularly including cardiac toxicity) [1], [2], [3], [4], [5], [6], [7], [8], [9], [10]. Developmental toxicity in fish occurs at very low doses and is easily studied in species that lay externally fertilized eggs with clear chorions, such as zebrafish (*Danio rerio*) [4], [11], [12]. As a result, fish species have been used extensively to study the mechanisms of developmental toxicity of dioxin and dioxin-like compounds [13], [14], [15], [16], [17], [18], [19], [20], [21], [22]. Many of these studies have focused on heart deformities as cardiac tissue is considered one of the most sensitive targets of dioxin toxicity in developing fish [4]. Some early hypotheses, such as that cytochrome P4501A (*cyp1a*) mediates dioxin-induced developmental cardiotoxicity, have not been supported by experimental evidence [23]. Therefore, various researchers have turned to multidimensional and high-throughput approaches such as gene expression microarrays to better understand the biological response to dioxin exposure early in development [24], [25], [26], [27].

Handley-Goldstone et al. [24] used a custom microarray on whole zebrafish that were 3 days post-fertilization, which had been dosed earlier in development. Carney et al. [25] examined RNA isolated from the heart as well as the whole fish at 4 independent time points (1, 2, 4, and 12 h post-exposure) from 3-day zebrafish dosed later in development (3 days post-fertilization). Yang et al. [26] examined RNA from whole zebrafish exposed either during the 1^st^ day, 2^nd^ day, or 5^th^ day post-fertilization. Our experimental design extends these previously published studies of dioxin-induced gene expression changes in zebrafish. We dosed whole embryos with dioxin for 1 h starting at 4 h post-fertilization, and then allowed them to develop to 1, 2, 3, 4, and 5 days post-fertilization, prior to harvesting samples for gene expression profiling.

We chose to analyze the resulting expression data in an interactome framework – a powerful environment for analysis of transcriptomic or other types of -omic data [28], [29]. Interactomes are global networks that map the interactions (or “links”) between network nodes (proteins and other biological actors such as genes, enhancer regions, cofactors etc.). For example, in the context of the canonical aryl hydrocarbon receptor (AHR)-based response to dioxin exposure, the AHR and ARNT proteins interact via a protein-protein interaction, and they both interact with *CYP1A* via a protein-DNA interaction due to the presence of AHR response elements in the promoter region of the *CYP1A* gene. Interactome-based approaches have particular promise in cases where subtle (small fold-change) alterations are observed in many genes, rather than large fold-changes in a few genes. Consistent with previous studies [24], [25], [30], [31], we found that dioxin exposure resulted in relatively low-magnitude changes in transcript levels for most affected genes. Furthermore, while enrichment analysis, such as in Gene Ontology categories, is an efficient way to interrogate the function of a given gene set of interest, interactome-based enrichment investigation offers a particularly powerful perspective on functional relations between the individual genes that were found to be differentially expressed. However, very few protein-protein or protein-DNA interactions have been identified for any fish species.

Therefore, we computationally inferred the zebrafish interactome using FunCoup [32] and InParanoid [33]. FunCoup is a tool that predicts Functional Coupling of genes or gene products based on information available in other species; we used information available in 8 eukaryotes. This information was transferred to zebrafish genes by using InParanoid, which has been shown to accurately identify orthologs [34], particularly co-orthologs (also called inparalogs – [35]) in species abounding in recent genome duplications, such as zebrafish [36]. The interactome served as the backbone for subsequent analysis of the microarray data, which was carried out using both previously-described and our own newly developed analysis toolsWe thus present a multifaceted analysis of the global gene expression response to dioxin exposure utilizing novel tools of network analysis. We made the zebrafish interactome and other resources used in the analysis public online (http://FunCoup.sbc.su.se/zfish.html). The interactome, incorporating the dioxin microarray data, is accessible both for graphic sub-network navigation and full download. The web site allows further autonomous analysis of the network, as querying is highly flexible via varying confidence level, evidence base, and network topology criteria.

## Results

We present below:

- a description of the generation and initial characterization of the microarray dataset obtained from zebrafish embryos after dioxin treatment;
- an overview of the interactome-based analysis that we carried out (parts III–V of Results);
- computational prediction of a zebrafish interactome via integration of multiple eukaryotic datasets;
- multipart analysis of experimental data from (I) incorporated into the interactome (III); and
- chronologically-organized highlights of the transcriptome changes and interactome dynamics caused by dioxin during early zebrafish development.

**Figure 1:**
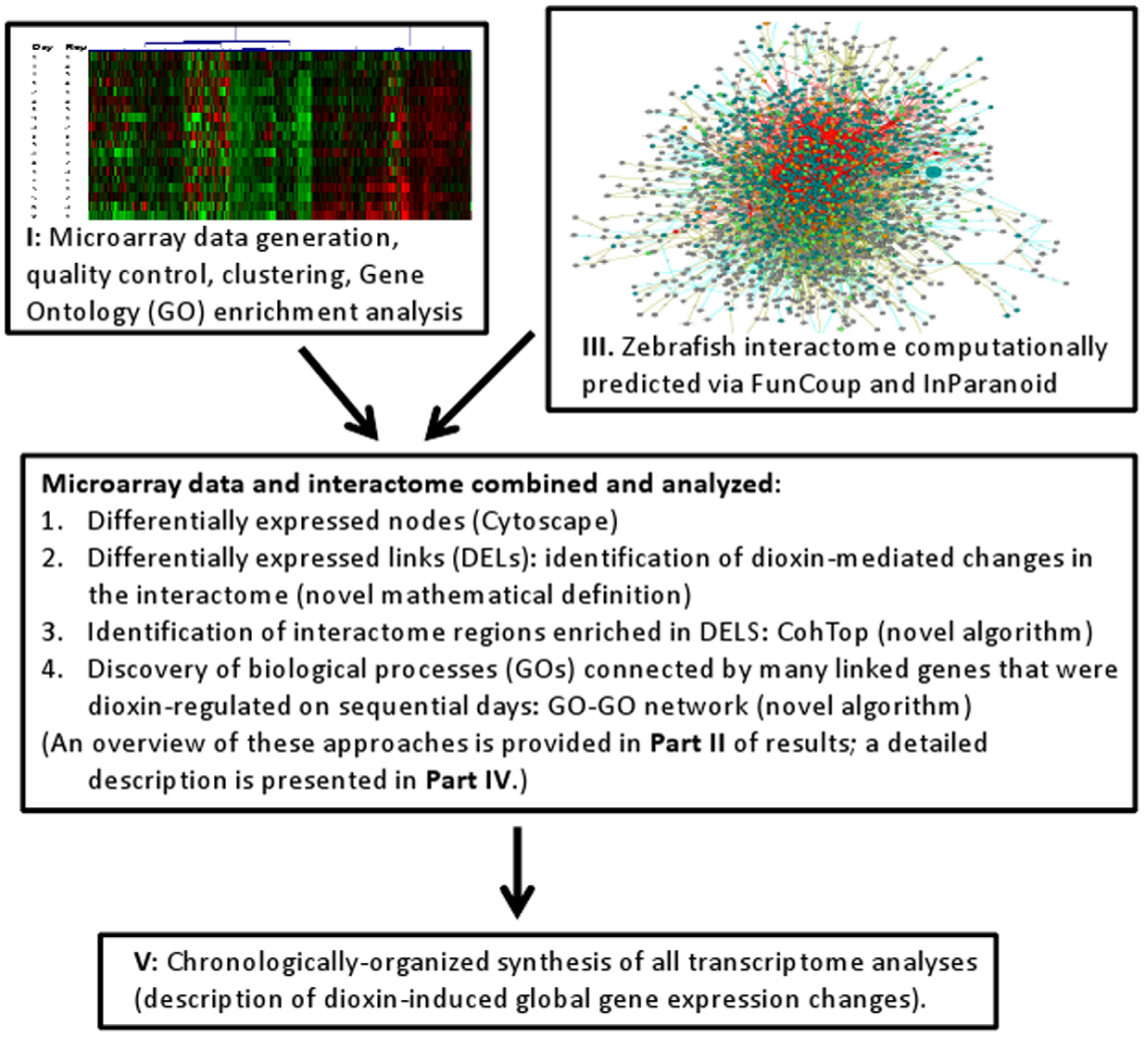
Overview of the workflow and organization of the manuscript.

Figure 1 illustrates the general workflow.

### I. Generation of an informative microarray dataset surrounding the onset of teratogenesis

Zebrafish embryos were dosed with 1 ng/mL dioxin for one hour at 4–5 h post-fertilization, and RNA was collected 1 day, 2, 3, 4, and 5 days later. While the eggs were rinsed thoroughly after exposure, it is important to bear in mind that dioxin is both highly lipophilic and very poorly metabolized, so a significant level of dioxin exposure undoubtedly continued throughout the experiment. This design was based on previous research showing that this dose [37] leads to toxicity first detectable at 2 days and more dramatic at 3, 4 and 5 days [12]. Thus, our time-course was designed to permit detection of gene expression changes preceding, including, and following the development of visible teratogenesis.

**Figure 2:**
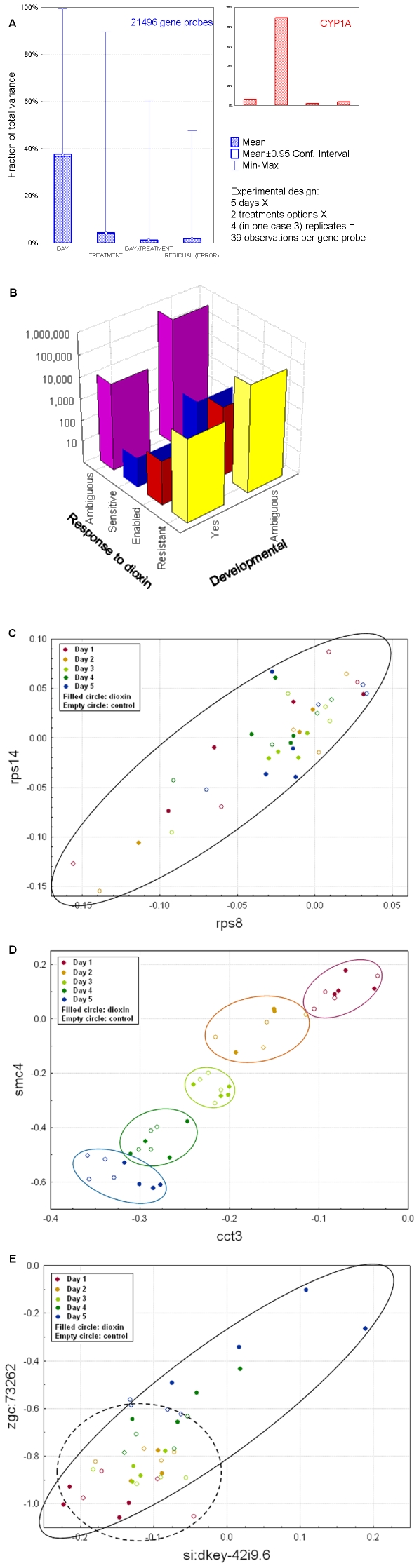
Experimental factors affecting differentially-expressed genes (A), occurrence of different kinds of differentially-expressed links (B), and examples of types of links (C–E). A. Fraction of variance [92] of transcriptional change for the whole set of analyzed transcripts (blue) and for *cyp1a* (red), associated with different experimental factors. While developmental changes dominated, dioxin modulated as many as 2009 genes at one or more time points in the experiment (detected by 2-way ANOVA on factors “DAY” and “TREATMENT”, applied to individual genes). 95% confidence intervals are too small to be seen in most cases. *cyp1a* regulation was largely treatment-dependent (the horizontal legends are identical to the whole set chart). B. Distribution of specific functional links inferred in the FunCoup network by analysis of microarray data. We discovered dioxin-enabled, dioxin-sensitive, and dioxin-resistant links, along with developmental stage-specific links. The majority of FunCoup links did not overlap with any of these categories, either because a gene was absent from the microarray chip, or because its transcription was not perturbed in the experimental conditions. The number of dioxin-sensitive links is smaller than the number of dioxin-enabled links, and both are much smaller than the number of dioxin-resistant links. Differentially expressed links were detected here via ANOVA (combined with Pearson correlation metric) by considering a third factor “GENE” and its interaction with “DAY” and “TREATMENT”. Note the logarithmic scale of the vertical axis. C, D, E. Visualization of specific link types. All 3 gene pairs were significantly co-expressed (PLC equaled 0.876, 0.946, and 0.754, respectively). Plot axes indicate relative transcript abundance of the genes (log_10_ ratio of the experimental value to the transcript abundance in the reference pool). C. For this correlation, the condition- and day-specific points were mixed randomly. The reason for the high co-expression of the two genes (both of which code for ribosomal proteins) was probably not associated with the parameters examined in our experiment, and this link would be characterized as “Ambiguous” rather than “Developmental” or dioxin-altered. D. The transcript levels for these two genes (coding for proteins involved in chromosome maintenance and protein folding) were strongly synchronized according to day, so the link was classified as “Developmental.” E. Co-expression was observed under the dioxin treatment, but absent in the control (PLC 0.9032 and 0.1286, respectively), so the link was classified as “dioxin-enabled”. These genes are uncharacterized—but now known to lose functional coupling after dioxin exposure. The differentiation between C, D, and E would not be possible without deeper variance decomposition in ANOVA. See Methods S1 for details.

We repeated this developmental time-course with and without dioxin exposure four times, approximately a week apart. Each replicate, therefore, constituted a truly independent experiment. The multiple replicates permitted calculation of in-group variability, crucial for the proper implementation of 2-way (transcription of individual genes) and 3-way (gene-gene links in the network) ANOVA. A summary of the variability is presented in Fig. 2A, as a heat map of significantly changed genes in Figure S1, and as pairwise global gene expression correlation coefficients for each individual microarray in Data File S1. Overall, the dataset of transcripts differentially expressed during development and in response to dioxin exposure was robust. The different days and treatment groups were correlated and clustered well. Furthermore, the measured expression levels of *cyp1a* (a well-studied and highly dioxin-induced gene) indicated 13-, 62-, 90- 71-, and 48-fold increases on days 1–5 respectively.

We analyzed lists of genes differentially expressed under different conditions (days after exposure, treatment, and their combinations) in a variety of ways. A list of altered genes identified as modulated by ANOVA analysis, along with the days on which they were differentially expressed and basic descriptions and GO annotations, is presented in Data File S2; a graphical presentation of the number of differentially-expressed genes at different fold-changes for each day is presented in Figure S2. We also carried out analyses using the well-established gene ontology enrichment analysis tool GOMiner [38], [39]. The GOMiner output files are available at http://funcoup.sbc.su.se/zfish_supplementary.html. However, due to the limited number of transcripts that were identified as differentially expressed at each time point and the limitations associated with the Fisher's Exact Test in this regard, the results of these analyses did not provide much indication of the biological processes being modulated in response to dioxin exposure. To more effectively derive biological insights from the transcriptomic response, we pursued an interactome-based analysis.

### II. Descriptive overview of interactome-based analysis

For our interactome-based analysis, we predicted the global network of functional coupling in zebrafish with data from other eukaryotes (Results Part III), and combined that interactome with microarray-based inference. We took three complementary approaches to analyze the resulting network, described in Part IV of Results. First, using the Cytoscape plugin jActiveModules [40], we identified network regions (sub-portions of the full network referred to as “modules”) enriched in differentially expressed, interconnected genes (nodes). This approach is based on altered *node properties* (i.e., dioxin-altered gene expression levels) given a fixed structure of network *interactions* (i.e., the interactome). Second, to complement this node-centric analysis, we clustered the network into modules based on *dioxin-altered interaction properties* given a fixed set of genes (interactome nodes). For this, we identified interactions between genes that

1. appeared or disappeared in a dioxin-dependent fashion (were dioxin-sensitive); or
2. were insensitive to dioxin exposure; and
3. were developmentally-regulated in the first five days after fertilization.

We found *top*ologically *coh*esive network clusters of such interactions with a novel software program named CohTop (publicly available at http://funcoup.sbc.su.se/zfish.html). Since this approach and tool are new, we dedicate Part IV A of Results to describing it. This approach allowed observation of alterations in the zebrafish interactome itself, as a function of dioxin exposure and development. Third, in another novel approach (Part IV B), we traced the temporal propagation of the dioxin-induced transcriptional signal through 5 days post-dioxin exposure via a network analysis where network nodes were generalized to Gene Ontology categories rather than genes.

### III. Zebrafish backbone interactome reconstructed via integration of data from eukaryotes

Several interactomes, either experimentally elucidated, computationally predicted, or their combinations, have been released for a number of eukaryotes [41]. However, no such resource has been described in a fish species, nor were any large-scale interaction datasets available. We employed the FunCoup resource [32], a set of programs and a custom collection of datasets informative regarding functional coupling (interactions), to generate a zebrafish interactome. FunCoup can predict interactomes of different confidence, i.e. interactomes in which each interaction requires greater or lesser amounts of supporting evidence (Fig. S3). Lower- and higher-confidence networks containing 5760 nodes with 99570 interactions and 3512 nodes with 32520 interactions are available as .sif files in Data files S3 and S4.

Because this approach has been described previously [32], we present most of the details of the interactome generation in Methods S1. Generally, FunCoup employs a deeply optimized technology of supervised learning to integrate multiple data sources including physical protein-protein and protein-DNA interactions, mRNA and protein expression, and many other pieces of weaker evidence into probabilistic scores that inform on likely interactions, or “functional couplings,” between genes. The identification of the orthologous genes required to transfer information from other species was carried out with InParanoid ([33]; see Methods S1 for details). The advantage of the InParanoid approach is that it considers evidence of functional coupling from *multiple* co-orthologs that might have emerged from genome duplications since the initial speciation event. Our interactome is based on data from eight eukaryotic species (*Homo sapiens*, *Mus musculus*, *Rattus norvegicus*, *Drosophila melanogaster*, *Caenorhabditis elegans*, *Saccharomyces cerevisiae*, *Arabidopsis thaliana* and zebrafish: Fig. S4). Our predicted networks were scale-free, as expected for gene interaction networks (Fig. S5).

### IV. Interactome-based analyses

As described above (Results part II), we carried out several independent types of analysis using the backbone interactome. Cytoscape analysis is not described separately since this is a well-known tool. Below, we describe analysis of dioxin-altered interactions (IV A) and temporal propagation of altered biological processes (IV B).

#### IV A. Extension to a dynamic interactome: some interactions exist only in the presence or absence of dioxin

Since the links composing the interactome were derived from many different kinds of data, they include interactions of many sorts (a common signaling pathway, metabolic process, protein complex etc.), and in any context (tissue, condition, or developmental stage). However, all interactions derived in such an integrative fashion will not occur under all conditions. Thus, a network analysis might be more efficient if it could identify and use interactions that emerge in a specific context. We were able to detect many such dioxin- or developmental stage-specific interactome links using our microarray data, and defined them as *differentially expressed links* (DELs), by analogy with differentially expressed genes. In the presence of dioxin, DELs were observed where mRNA expression correlations appeared (*dioxin-enabled*) or disappeared (*dioxin-sensitive*). In our experiment, the cutoff at Pearson's linear correlation coefficient (PLC)>0.75 defined a correlation over the whole profile of 39 expression values as significant (*p_α_*<0.01; *p_FDR_*<0.16). To detect DELs, we calculated PLC on each half of the total expression profile, i.e. either in the presence or absence of dioxin, and performed 3-way ANOVA with factors “GENE”, “DAY”, and “TREATMENT” (Methods S1). With additional ANOVA-based criteria, we selected interactions that reflected functional changes in the network. Figure 2C and 2E compare expression correlated in a dioxin-irrelevant fashion (2C) with that in a dioxin-enabled link (2E). For comparison, Fig. 2D shows a link where expression dynamics were developmentally synchronous, yet unchanged by exposure to dioxin. Simple correlation analysis without ANOVA criteria would not have permitted this important distinction to be made. For such a link to be significant, we required its *overlap with a link predicted by FunCoup from data integration*. A rough estimate of the false discovery rate for dioxin-enabled and dioxin-sensitive DELs, when combined with FunCoup links, was 11% (Methods S1). The DEL fraction in the interactome – 1.1% of all FunCoup links (Fig. 2B) – was an order of magnitude lower than that of differentially expressed genes, potentially permitting a more focused analysis. Although dioxin-enabled links were more abundant than dioxin-sensitive interactions, the number of dioxin-*resistant* interactions (i.e., those occurring irrespective of dioxin) was an order of magnitude larger still (Fig. 2B).

**Figure 3:**
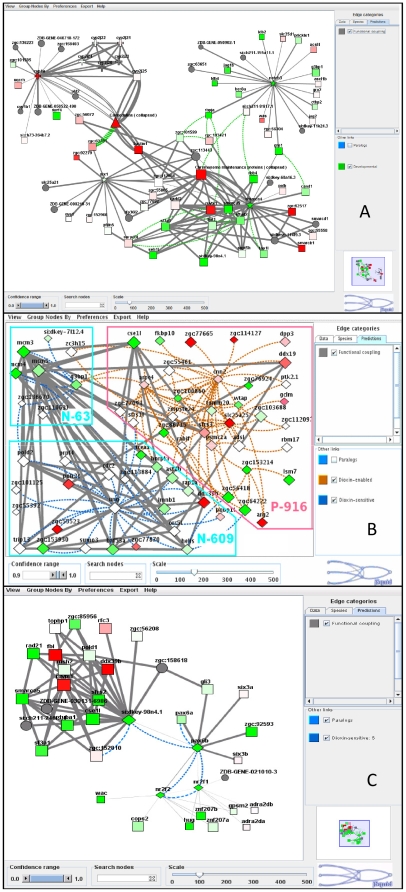
FunCoup sub-networks with genes and links affected by dioxin. A. Network region around the originally (on day 1) altered CYP1A includes links to gene groups altered later (as discovered with jActiveModules and temporal GO relation analyses): calmodulins (triangles), members of heme biosynthesis (crosses), and neuronal development (stars) pathways. B. Network region with three adjoining clusters enriched in differentially expressed links. *cnn2* (calponin 2) is co-expressed with many surrounding genes only after dioxin exposure (cluster P-916), while *ung* (uracil DNA glycosylase; cluster N-609), on the contrary, is normally co-expressed with its network neighbors but not after dioxin exposure. Meanwhile, several “border” genes lost functional coupling to genes from clusters N-609 and N-63, while gaining coupling to cluster P-916 genes, after dioxin exposure. The clusters are named P-* when containing positive (dioxin-enabled) links, N-* for negative (dioxin-sensitive), or PN-* for both positive and negative links. C. Members of cluster N-632 include DNA/RNA processing, cell division, and embryonic forebrain patterning genes. Interactions: Grey, FunCoup links at confidence FBS>9 (Fig. B) and FBS>3 (elsewhere) (every blue and brown link must overlap with a FunCoup link at FBS>3). For every sub-network, only a sub-set of links was retrieved, filtering for confidence and relevance to query genes with algorithm (1) (see description at http://funcoup.sbc.su.se/algo.html#noislets). Brown, clustered (co-expression + FunCoup) links *caused* by dioxin treatment; Blue, clustered (co-expression + FunCoup) links *disabled* by dioxin treatment; Green, developmental-stage specific co-expression. Color lines at A indicate data types evident of functional coupling and are explained in right panel of the screenshot. Nodes: Diamonds: members of respective clusters (i.e., genes participating in a DEL). Squares: other genes with measured expression in the course of the experiment; Circles: other genes having evidence from orthologs in FunCoup but for which expression data was lacking in our experiment. Shades of red and green, up- and down-regulation (main factor “DIOXIN TREATMENT”) with dioxin, respectively; genes missing microarray data colored grey. All modules can be found and manipulated at http://funcoup.sbc.su.se/zfish_supplementary.html, and GO enrichment analysis of modules significantly enriched in at least one GO biological process are presented in Data File S5. Both clusters and individual genes can be accessed at http://funcoup.sbc.su.se/zfish.html. See Methods S1 for details of link inference and other analysis.

We next identified network modules significantly enriched in DELs using an in-house clustering program (CohTop; see Methods S1). Thus, dioxin-enabled modules are enriched in links that appeared only in the treated condition (e.g., Fig. 2E), and dioxin-sensitive modules were enriched in links observed only in the normal condition (i.e., were vulnerable to disruption by dioxin). We defined modules enriched in dioxin-enabled, -sensitive, or mixture of both types. We identified 151 dioxin-enabled modules, 142 dioxin-sensitive modules, and 186 modules enriched in both dioxin-enabled and dioxin-sensitive links. These modules ranged in size from only a few nodes to dozens (maximally 30 for dioxin-sensitive modules, 86 for dioxin-enabled modules, and 122 for mixed dioxin-sensitive and-enabled). For a complete list of all DEL-enriched modules presented as interactive clusters, go to http://funcoup.sbc.su.se/zfish_supplementary.html. The modules on the website identify core nodes (those participating in a DEL) as diamonds, but also show their immediate network neighbors. The website contains rich functionality for graphical and tabular analysis and allows components of interaction evidence to be aligned and studied both within and across species. Data File S5 contains GO enrichment analysis of modules that were significantly enriched in at least one GO biological process. While not a focus of this manuscript, we also defined developmental links: edges connecting genes co-expressed synchronously in the course of development (exemplified at Fig. 3A; see Methods S1 for details of the calculation).

A variety of patterns in dioxin-enabled and dioxin-sensitive modules could be observed. For example, some clusters of genes are correlated in expression only after dioxin exposure. *cnn2* (calponin 2) in cluster P-916 (Fig. 3B) gains functional coupling to 23 surrounding genes only after dioxin exposure. Since *cnn2* is likely involved in heart muscle structure and function via actin interactions, this dioxin-specific co-expression could be related to heart abnormalities caused by dioxin. Another pattern of interest that we observed is that dioxin-enabled and -sensitive modules often adjoined each other, highlighting genes that delineate a border between the clusters of genes whose pattern of interaction is impacted by dioxin. For instance, after dioxin exposure, *g3bp1* (GTPase activating protein (SH3 domain) binding protein 1) loses its functional coupling with chromosome replication genes *mcm3*, *mcm4*, and *mcm5* (cluster N-63), but gains functional coupling with actin-associated genes *cnn2* and *arpc4* (cluster P-916) (Fig. 3B). Such genes might potentially switch functions after dioxin exposure, or insulate certain functions from dioxin's toxicity.

#### IV B. Temporal propagation of transcriptome alterations

*Many network interactions connect the few transcripts altered on day 1 and the many altered on later days.* In this part of the analysis, we employed the backbone FunCoup network with transcripts altered in our microarray.

We found that there was a statistically significant enrichment in links between transcripts that were dioxin-regulated earlier and transcripts regulated later in the course of the experiment. This suggested that signals were propagated along network routes from the initially affected (on day 1) genes and network regions towards network regions that were perturbed later.

Specifically, over 2000 genes were up- or down-regulated by dioxin on at least one of the days of the experiment (defined as significance of the contrast “particular day X dioxin treatment” at *p_α_*<0.01), but only 81 of them were altered on day 1. For comparison, expression of 5850 genes was developmentally-regulated (i.e., developmental stage-specific and irrespective of dioxin exposure: main effect “day” at *p_α_*<0.01). The 81 “day 1” genes had 8755 interactions in the FunCoup network at FBS>3. They directly contacted 45.9% of the 1946 genes modulated by dioxin at later time points, while only 8.7% would be expected to be contacted by chance. The dioxin-altered genes exhibited higher connectivity: the average number of interactions per gene from this group was 79.5, compared to 50.1 for non-altered genes (1-way ANOVA, *p_α_*<10^−6^). We provide a list of these genes with interactions to their network environment at http://funcoup.sbc.su.se/zfish_day1.html. Data File S6 details the number of dioxin-regulated genes that are linked to each of the genes altered on day 1, while Data File S7 lists respective links individually. As an example, *cyp1a*, altered on day 1, was linked to genes of the calmodulin family altered from day 2 forward (Fig. 3A).

To gain a better perspective on what this temporal pattern in enriched connections between dioxin-modulated genes might mean, we analyzed Gene Ontology categories (GOs) associated with the connected nodes. These results are given below.

*Generalized propagation analysis reveals temporal relations between dioxin-altered GOs.* Interactome links indicate a general likelihood of genes/proteins to be functionally coupled, but do not highlight defined temporal directionality in those connections. Causal relations can be suggested by examining temporal changes: if information associated with entity *A* at time point *t* helps to define the state of entity *B* at time point (*t*+1), then a causal relation A→B might be inferred [42]. However, the 24 h intervals of our experiment would not fit the time scale of functional communications between most genes, and the statistical power from only 5 time points would be too low for robust analysis. To overcome this limitation, we generalized the network of individual genes to a network of GO “biological process” categories as described below. At this broader scale, relations between nodes (now GO biological processes) are more statistically reliable; links reflect statistically enriched temporal connections between multiple genes of one node with multiple genes of another. Thus, this “GO-GO network” highlights flow between GO biological processes affected by dioxin on different days (*meta-flow*). We refer to the links as GO-GO interactions.

**Figure 4:**
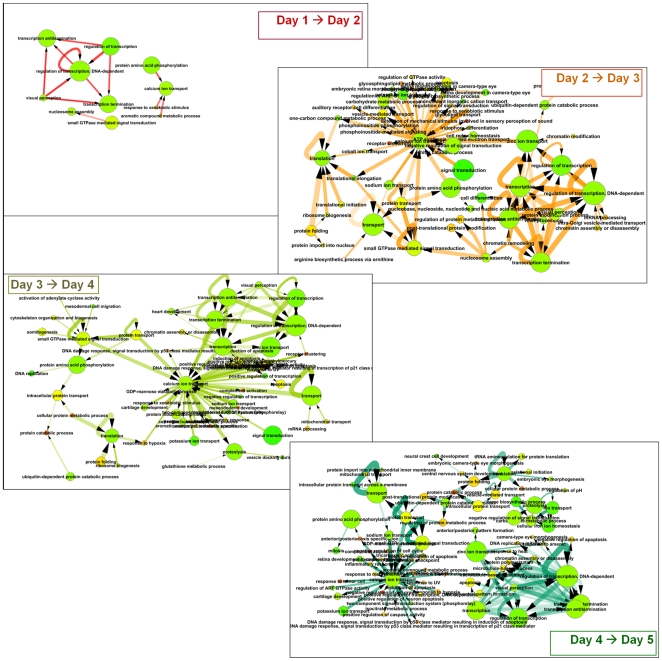
Network of biological processes based on genes dioxin-altered on sequential days. Nodes of the network are defined as GO “biological process” categories that include one or more differentially expressed genes in the course of the experiment. Thus if a GO category is enriched on day 1 in differentially expressed genes, and linked by an unexpectedly high number of FunCoup interactions to a second GO category that on day 2 is enriched in differentially expressed genes, we infer that this temporal relationship is indicative of causality. Network interactions (edges) represent the sum of interactions between the gene members of the two connected GO categories. Node color represents the fraction of the genes in that node that are regulated by dioxin on any day (green is low, red is high). Edge thickness and opacity represent the number of gene-gene links between two categories and χ^2^ enrichment score for the likelihood that this pair of categories is enriched in links, respectively. Edge color and arrows show timing of differential expression between gene-gene pairs in respective GO categories.

Dioxin-altered genes in individual gene-gene interactions were labeled with days when the participating genes were first detected as differentially expressed. The inferred GO-GO interactions then fell into 2 classes: ones where both genes were first altered on the same day, and those where expression of one of the genes changed earlier than the other. By only using the latter class, we could augment the analysis with a temporal dimension. Thus, if there were a significant number of genes of GO category *X* first altered on day *d* interacting with genes in GO category *Y* first altered on day (*d*+1), then we could hypothesize a causative relation (meta-flow) *X*→*Y*. Limiting the output to only *enriched* GO-GO connections, i.e. ones with significantly more gene-gene interactions (given that both genes were dioxin-regulated) than expected by chance, allowed us to focus on the major tendencies of propagation of toxicity and organismal response to it. Compared to the individual category enrichment, this approach yielded a much richer analysis for interpretation. The dynamic picture of day-by-day meta-flow is presented in Figure 4. The combined map of all flows, as well as same-day networks, are offered as Figure S6 and Data File S8 (a manipulable Cytoscape session file).

### V. Chronological analysis of the interactome altered by dioxin exposure

The tools described above enabled flexible and deep monitoring of dioxin-induced changes in the transcriptome and interactome. The following day-by-day analysis highlights results of particular interest. More extensive results can be found at the website and in the supplementary files. Cytoscape session (.cys) files containing the day-by-day jActiveModules (jAM) outputs are available at http://funcoup.sbc.su.se/zfish_supplementary.html. Data File S9 shows a GO enrichment analysis of all differentially expressed genes (by ANOVA) for all possible contrasts (treatment and day combinations; see Methods S1). Data File S10 shows a GO enrichment analysis of all dioxin-modulated genes, by day.

#### Day 1 (20 h after exposure, 24 h post-fertilization)

We detected only 81 dioxin-regulated genes on day 1 (ANOVA, *p_α_*<0.01). 55 of them were altered on each of the 5 days, supporting the reliability of the day 1 list (dioxin is a very slowly-metabolized compound, so a single dose leads to a long-term exposure).

On day 1, we identified only one large and high-scoring jActiveModules (jAM) neighborhood (i.e., subnetwork statistically enriched in dioxin-regulated transcripts). *cyp1a*, the classic biomarker of dioxin exposure, was highly (13-fold) upregulated at this stage, but was not a part of the most-altered neighborhood. This single jAM neighborhood was characterized by a fairly large number of transcripts that were in most cases expressed at slightly (<2-fold) rather than highly (>2-fold) altered levels (Fig. S7). Among the GO categories significantly overrepresented in this module were proteolysis, stress response, DNA metabolism, regulation of cell cycle, embryonic morphogenesis, and glycolysis. However, many of the most-altered transcripts in the highest-scoring neighborhood for day 1 were poorly annotated. Conversely, many genes included in this neighborhood were not present on the microarray. Therefore, we manually inspected the genes that comprised this neighborhood. Of particular interest was the large number of transcription factors altered (as identified by Rosetta Resolver analysis), many of which are known to be involved in embryonic morphogenesis and/or stress response: *fos*, *pax2a*, *foxd5*, *foxi1*, and *ubtf*. *cdk5*, a cyclin-dependent kinase involved in neuronal maturation, was down-regulated, which is interesting as multiple neuronal maturation-related transcripts were altered at later time-points.

Analysis of the full list (i.e., regardless of network topology) of dioxin-modulated (as defined by ANOVA, *p_α_*<0.01 for main effect of dioxin treatment) genes for day 1 yielded only one enriched GO biological process: iron homeostasis (Data File S10). This enrichment list consisted of CYP1A together with 3 other genes (cytochrome P450 *cyp11a1*, an embryonic hemoglobin *hbbe2*, and *zgc:109934*). The expression of different genes involved in iron homeostasis changed throughout the time course; however, iron homeostasis as a GO term was found to be significantly enriched on multiple days.

#### Day 1 to Day 2 transition

An examination of meta-flow of GO categories between genes regulated on day 1 and those first altered on day 2 (Fig. 4) suggested a cascade initiated by changes in transcription factors and xenobiotic metabolism genes and leading to genes involved in calcium ion transport, protein metabolism and folding, and transcriptional regulation. Relatively few calcium-related proteins were identified on day 1; two examples were *cab39* and *heg*. While the CYP1A neighborhood itself was not heavily altered on day 1, it had metaflow GO category connections “day 1→day 2” to “calcium ion transport”. In particular, although none of calmodulins were dioxin-regulated on day 1, eight of them were strongly over-expressed later, beginning on day 2.

As described above, many genes differentially expressed on day 1 code for transcription factors and proteins involved in morphogenesis. 144 transcription factors were eventually altered during the experiment. *vsx2* (*chx10*) was down-regulated and is particularly interesting in that it shares a number of network neighbors with calmodulins and is also functionally coupled to the transcription factor *yy1* (FunCoup-linked to erythropoietin, a dioxin-perturbed member of the hypoxia pathway; see an interactive network of the latter at http://funcoup.sbc.su.se/zfish_supplementary.html) and kinases *junb*, *junbl* (both up-regulated on days 4 and 5) and to estrogen receptor *esr1* (downregulated from day 2 forward). *vsx2* is a homeobox gene involved in retinal development that belongs to the “visual perception” category, and was the first to alter. Other members of this pathway were affected later in the course of observation.

Further reinforcing the pattern of a time-dependent propagation of altered gene expression, a number of RAS signaling proteins altered on day 1 were linked to nucleosomal proteins (mostly histones) altered on day 2. The alteration of protein folding activity (31 out of 96 currently known zebrafish genes in this GO category were affected in total) was initiated on day 1 with *dnaja2l* and *ppih*.

#### Day 2

**Figure 5:**
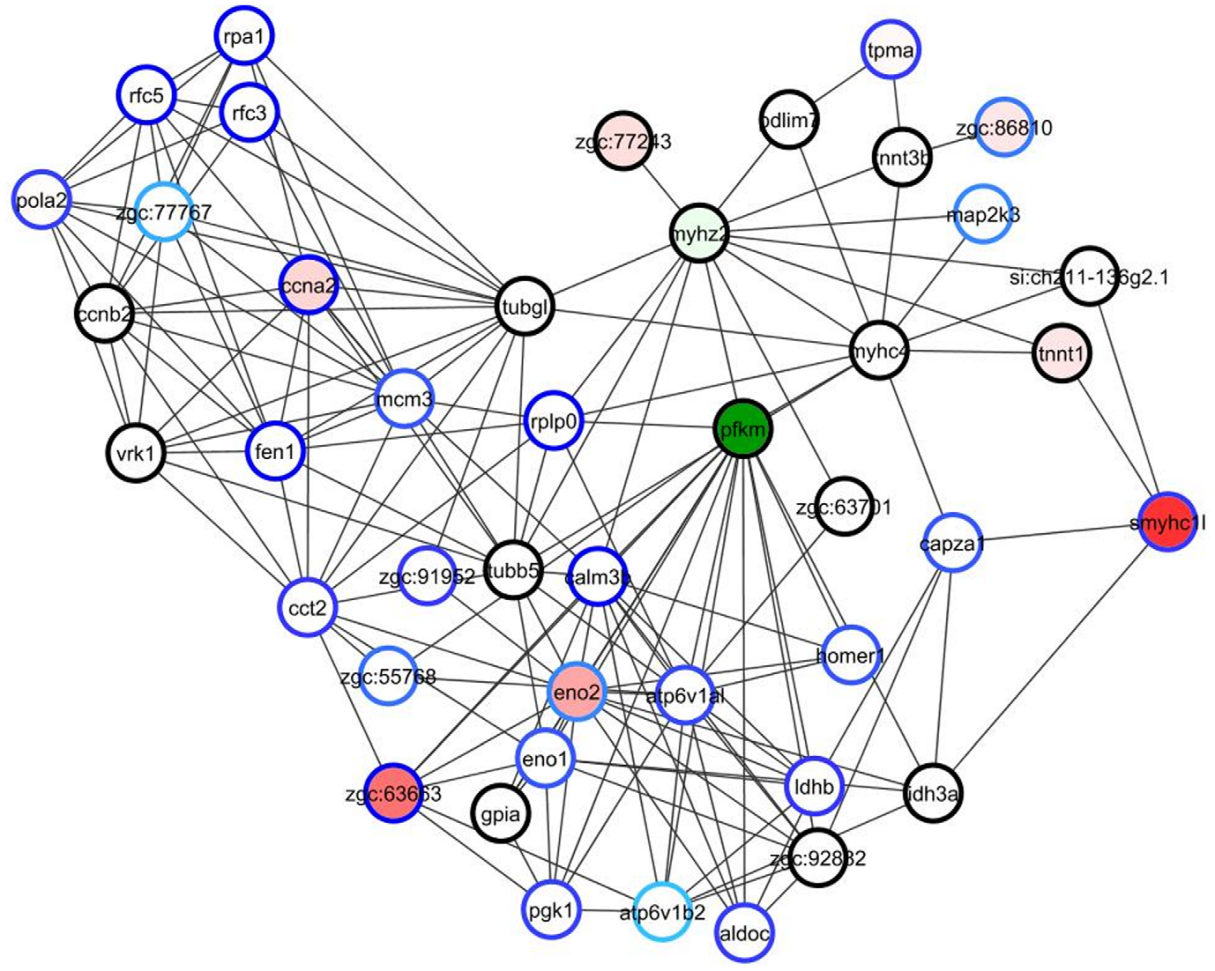
Many genes in an altered (jActiveModules) neighborhood identified on Day 2 are involved in glycolysis. DNA repair and maintenance as well as muscle-related cytoskeletal proteins are also part of this module. Red node color indicates upregulation and green indicates downregulation, with the faintest coloration indicating a 1.3-fold change and darker coloration indicating greater change. Node border color indicates significance; the faintest blue indicates *p_α_* = 0.05, with darker blue indicating greater statistical significance (by Rosetta Resolver).

Multiple high-scoring altered neighborhoods appeared on day 2, and at this time the top neighborhood identified by jAM did include *cyp1a*. Several of the GO biological processes that were altered on day 1 were now affected more strongly, both in terms of number of genes altered and the magnitudes of their expression differences. For example, dioxin-induced processes that exhibited changes in many more genes included the stress response, embryonic morphogenesis, gene transcription, protein metabolism, and DNA metabolic processes including proteins involved in DNA replication and/or repair. Glycolytic energy metabolism remained altered, but differently than on day 1 (Fig. 5). Transcripts for most other proteins involved in glycolysis showed smaller-fold but significant changes in expression, but the directions of the changes were mixed. For example, while phosphofructokinase (*pfkm*, a critical control point for glycolysis) was upregulated 2-fold on day 1 by dioxin, it was down-regulated later: 2.5-fold on day 2 and increasingly suppressed until day 5, and then returned to normal. The set of embryonic morphogenesis genes altered on day 2 differed from those of day 1, now coding for proteins involved in other processes such as axon formation and tissue regeneration (*cxcr4b*, *cdh2*, *robo2*, *gap43*). Genes involved in heme synthesis were up-regulated, while many (but not all) heme/porphyrin-containing genes were down-regulated. Other GO processes identified as newly altered on day 2 were chromosome organization and biogenesis, heart development, generation of neurons, and tissue regeneration.

#### Day 2 to Day 3 transition

Day 2 was marked by the most significant daily increase of linkage from transcription factors altered on day 2 towards others altered later. The most dramatic central node of the day 2→day 3 GO network was calcium ion transport (Fig. 4), which became connected to multiple biological processes, such as signal transduction, response to hypoxia (chaperonin *cct4* and *arnt2*), response to xenobiotic stimulus (now *cyp3a65* joined *cyp1a*), retinal and auditory receptor development, and others.

#### Day 3

On day 3, the response of stress-related GO categories was more noticeable than on days 1 or 2, and included genes related to xenobiotic metabolism- and the AHR pathway (*gpx4a*, *cyp3a65*, *ahr2*, *cyp1a*, and others) and DNA damage/metabolism (*rpa2*, *pcna*, *gtf2h3*, *rad23b*). *fos* and *junbl* were also up-regulated, and connected a small cluster composed of altered extracellular matrix (fibronectin and collagen) genes (Fig. S8). Alteration of protein folding and catabolism, calcium ion transport, heme biosynthesis, and glycolysis-related genes did not differ from that on day 2. Newly-affected biological processes included protein translation and cell cycle. A variety of developmental and morphogenesis genes were altered, with a prominent addition being *dbx1b*, a brain homeobox gene that was more than 8-fold down-regulated by dioxin.

#### Day 3 to Day 4 transition

Calcium ion transport was again a central node in the day3→4 GO propagation network, but now linked to processes specific to day 4. Many embryonic development categories (mesendoderm development, cartilage development, anterior/posterior axis specification) altered on day 4 were enriched in interactions to genes altered on day 3 in the calcium ion transport category. Similarly, the GO network suggests a strong flow from calcium ion transport genes of day 3 to many stress-response genes of day 4 (positive regulation of apoptosis, DNA damage checkpoint, response to UV, response to xenobiotic stimulus, negative regulation of cell cycle and cell growth). Calcium ion transport genes also remained linked to important transcriptional regulators, and impacted small GTPase-mediated signal transduction of day 4.

#### Day 4

The GO biological processes associated with many of the most-altered portions of our interactome identified by jAM on day 4 often overlapped with those of day 3. In particular, stress response, heme synthesis, protein catabolism, translation and folding, glycolysis, and cell cycle remained altered. Iron homeostasis emerged as an independent category, as did hemopoeisis. Considering that these samples were taken 4 days after dosing, and thus after the onset of outright teratogenesis, the changes observed in terms of number of genes and fold-change were relatively modest, even among the most altered jAM network neighborhoods (for example, see top modules for both interactomes for day 4 at http://funcoup.sbc.su.se/zfish_supplementary.html).

#### Day 4 to Day 5 transition

The “calcium ion transport” node remained central and was now linked to a large group of relatively compact and highly enriched GO categories related to mitosis, DNA damage, and apoptosis – a feature likely manifesting a later stage of the stress response. Interestingly, a distinct group of cell division category genes, such as tubulins, was connected to transcription regulation gene sets rather than to calcium ion transport. The ion channel proteins of the transport category that was significant at earlier days were now complemented with several ADP ribosylation factors and RAB proteins. Altered transcription regulation genes, of both general and specific GO categories, were heavily interconnected. Linkage between signaling and embryonic development categories remained noticeable. Novel categories included mitochondrial transport and complement activation (Data File S8, day 4→day 5 subnetwork).

#### Day 5

By far the most differentially expressed genes were identified on day 5. Nonetheless, many dioxin-perturbed GO biological processes of day 5 had already been identified as altered on earlier days. Protein folding genes were upregulated in the dioxin-exposed samples, as were apoptosis, stress response, and DNA damage response genes. Altered responses included a return of *pfkm* expression levels to normal, and the disappearance of glycolysis as an altered biological process. Actin polymerization genes were upregulated, and multiple developmental trajectories were altered more strongly or for the first time at day 5, including neuronal and eye development, cardiac cell differentiation (which was especially marked), and keratin. There were also alterations in immune system genes and the FOS/JUN and NFKB stress response pathways.

#### Dioxin-altered interactions (all days)

The appearance and disappearance of interactions after dioxin exposure is particularly interesting as it be informative regarding the enabling of circuits that are caused by, adaptive to, or otherwise associated with teratogenesis, as well as the disabling of circuits whose function is typical for normal development. There were relatively few of these dioxin-altered interactions, and we chose to focus on modules enriched in such interactions on the assumption that the groupings would be both statistically significant and biologically relevant.

The network modules found with CohTop typically had much higher GO enrichment scores than did lists of individual differentially expressed genes. This suggests that modules enriched in dioxin-altered interactions often comprised highly coherent protein sets. Across all the discovered modules, the most significantly enriched GO categories were DNA, RNA, ATP, and GTP binding (e.g. *arf*5 and, less significantly, other ADP-ribosylation factors), as well as categories that characterize proteasomal, ubiquitylation, and chaperonin proteins (Data File S5). This last category was also identified by other tools as dioxin-altered.

Numerous hypotheses emerge from a close examination of the dioxin-enabled and –sensitive links and the modules they compose. As an example, cluster N-632 (Fig. 3C) illustrates a dioxin-dependent loss of interactions. Co-expression links between two transcription factors (*pax6a* and *pax6b*), two nuclear receptors (*nr2f1* and *nr2f2*), topoisomerase 2B (*si:dkey-98n4.1*), and the nucleolin *zgc:152810* disappeared after dioxin treatment. This suggests that a functional relationship that existed between the *pax6* and *nr2f* transcription factor networks was disrupted, and that coordination between these transcription-related networks and the associated (in the absence of dioxin) adjacent DNA replication and repair/RNA processing/cell division network was altered by dioxin.

Calmodulins were involved in both dioxin-sensitive and dioxin-enabled modules in a sub-network comprising 140 proteins (six modules listed at http://funcoup.sbc.su.se/zfish_calmodulin.html). This supported the identification by other tools (above) of calcium metabolism-related genes as highly altered by dioxin exposure.

Finally, it was striking that the CohTop clustering did not identify any “day 1” dioxin-enabled/-sensitive modules. In total, 33 of the 81 genes differentially expressed on day 1 participated in such dioxin-altered interactions. 16 genes were found in the dioxin-enabled modules and 2 in dioxin-sensitive modules, but no module contained more than a single differentially expressed gene of day 1. In other words, the genes initially modulated by dioxin were relatively isolated, and as a result the network was minimally perturbed on day 1, but much more perturbed on later days. This observation supports the hypothesis that dioxin initially affects a small number of genes, and that this signal is then further propagated on a longer time scale – days rather than hours.

## Discussion

In this work, we generated and analyzed microarray data related to dioxin embryonic toxicity using a new interactome and novel analysis tools. This enabled us to review the progression of dioxin-induced changes in gene expression and the network structure itself in the zebrafish at the most dioxin-vulnerable stage of its development. The efficiency of our analysis of dioxin-altered global gene expression was enhanced by the interactome approach, as the network-based analysis tools were superior to their single-gene analogs in terms of both statistical power and biological interpretability. A variety of interesting biological hypotheses result from our analysis.

Despite the marked induction of the transcript for the classic dioxin biomarker *cyp1a*, the earliest response to dioxin at a network level was not strongest in the neighborhood of *cyp1a*. Rather, on day 1, the most changed neighborhoods were those associated with lysosomal proteolysis, DNA metabolism, transcription, cell cycle progression, glycolysis, and embryonic morphogenesis.

In general, the genes that were significantly changed on day one were scattered throughout the interactome and did not comprise a single network neighborhood. However, our novel analytic approach demonstrated that the small focal responses observed on day 1, including stress-responsive and xenobiotic-responsive (e.g., *cyp1a*-associated) biological processes, were linked under the requirement of statistical significance to larger neighborhood responses at later days. These linkages were also extensive: the genes that were dioxin-regulated on day 1 were linked (FunCoup) to >45% of all of the genes that were dioxin-regulated at later days. This result suggests a time-dependent propagation of the dioxin-regulated transcriptomic signal: we hypothesize that the changes in gene expression that occurred on day 1 cause some of the gene expression changes that occur on later days, which underlie the teratogenic effect of dioxin. Since dioxin is recalcitrant to metabolism, the temporal progression of modulation of linked genes may relate as well in part to sustained dioxin exposure (*cyp1a* induction was maintained at a high level even at day 5). Many of these genes that are regulated by dioxin appear to be related to developmental pathways known to be perturbed by dioxin (e.g., *chx10*, *dnaja2l*, *cyp11a1*, *ppih*). A full discussion relating these genes to the molecular mechanisms of dioxin activity is not warranted without additional experiments. However, *cyp1a* particularly merits a discussion: its central role is surprising when compared to previous results.

While a 2003 study suggested that knock down of *cyp1a* expression in *D. rerio* reduced the developmental effects of dioxin exposure [43], other studies indicate that *cyp1a* expression is not required for the teratogenicity of dioxin in *D. rerio* [23], and that *cyp1a* expression and/or activity may even be protective in *D. rerio* and other fish species when exposed to other AHR agonists [44], [45], [46], [47]. Mammalian CYP1A1 and CYP1A2 generally appear to be either protective or irrelevant to dioxin teratogenicity [48]. However, *Cyp1a1(−/−)* mice were protected against high-dose acute dioxin-mediated toxicity, including uroporphyria [49]. Our findings suggest that some important aspects of dioxin-mediated developmental toxicity may be influenced by CYP1A induction, even if outright induction of deformities during development is not.

Multiple cytochrome P450s, including CYP1A but also CYP2, CYP3, and CYP4 family enzymes, participate in a number of pathways that produce and process bioactive molecules derived from fatty acids, e.g. eicosanoids. Many of these molecules have signaling roles both in endogenous processes and in response to xenobiotic exposure, and there is growing evidence that perturbation of these pathways is important in the toxicity of AHR ligands [50], [51], [52]. Furthermore, many processes regulated by eicosanoids were altered in our data. Therefore, we carried out a directed investigation of whether the pathways “Arachidonic acid metabolism,” “Steroid biosynthesis,” “C21-Steroid hormone metabolism,” and “Retinol metabolism” (as annotated in the *D. rerio* version of KEGG pathway database) were altered in our microarray data. Our network included many members of these pathways, but did not show any significant dioxin-related dynamics, either independently or in relation to CYP1A. On the contrary, we observed multiple “dioxin-resistant” links in these pathways. Of course, it is possible that this lack of an effect is a reflection of incomplete understanding of all of the relevant proteins and interactions. There are hundreds of eicosanoids, and their metabolism is far from fully elucidated. Therefore, key P450s (and other genes) may not be incorporated into our interactome, or evidence may be lacking for their respective interactions. Some were also not included on the arrays, such as the important AHR-responsive gene *cyp1b1*.

While heart deformities have been a major focus of research into developmental dioxin toxicity in fish and birds, other effects are highlighted by our results. Genes associated with embryonic morphogenesis were altered throughout. The number of affected genes, as well as respective processes and tissues, increased in the course of the experiment. The transcriptional dysregulation of genes involved in heart and muscle development is consistent with previous data suggesting that some of the heart teratogenesis may be expression-driven [24], [25]. Interestingly, genes involved in neuronal maturation were also altered, suggesting neuronal alterations in dioxin-exposed zebrafish. Such an effect has been reported in a variety of species including humans [53], [54], [55], but only rarely in fish [56]. Eye/retina development was affected as well, beginning with the homeobox gene *vsx2* and expanding to additional genes. While eye deformations other than slower eye pigmentation and development have not been a highlight of fish studies, they have been noted in other model animals [53], [57], [58], [59], [60], [61]. Thus, our results suggest that dioxin-mediated alteration of these processes is conserved in zebrafish, and can therefore be efficiently studied in this powerful model organism.

Perhaps the most striking trend in the network meta-flow between GO categories was the central position of calcium ion transport. This supports previous observations of a role for altered calcium homeostasis in mediating dioxin toxicity at very early time-points [62], [63]. While we did not examine such time-points, our results support a critical role of calcium ion homeostasis in propagating the teratogenicity of dioxin over longer time-courses. Indeed, dioxin has been shown to affect other calcium-related proteins and signaling pathways [64], [65].

We identified a strong alteration in regulation of genes related to iron homeostasis, including multiple cytochrome P450s, heme biogenesis and metabolism proteins, and others. Thus dioxin's toxic effects may relate to dysregulation of iron metabolism in fish. This idea is supported by the findings of AHR-dependent heme synthesis dysregulation in adult mice [66], and of porphyria in dioxin-exposed mammals and birds, including humans [49], [53], [67], [68]. Additionally, Belair *et al.* [69] found that in *Danio rerio* early embryonic exposure to dioxin interfered with the switch from embryonic to adult erythropoiesis and caused anemia, and Hahn and Chandran [70] showed that heme metabolism could be affected by dioxin in a fish cell line.

Multiple dioxin-altered mitochondria-related biological processes included calcium and iron/heme metabolism, apoptosis, mitochondrial transport, and glycolysis. A significant body of work describes dioxin-induced mitochondrial toxicity [71], [72], [73], [74]. However, while much of the previous work focused on induction of mitochondrial oxidative stress by dioxin, we did not find a strong transcriptional signal indicative of such a stress, despite the deliberate inclusion of a manually-assembled antioxidant response element network into the interactome analysis. This is also surprising since previous studies have shown that dioxin and related planar halogenated aromatic compounds can cause oxidative stress in various tissues or subcellular fractions, in particular microsomes [72], [75], [76], [77], [78], [79]. It is possible that such generation of oxidative stress is restricted in terms of relevant cell types, and thus the transcriptomic signal was too dilute in our whole-organism extraction to be detected. There may also be species-specific differences in terms of antioxidant defenses [80], although the studies cited above include both mammals and fishes. Another possible limitation is technical—the array we used does not include probes for any mitochondrial DNA-encoded genes, so any alterations in their levels will have gone undetected.

We identified several dioxin-altered biological processes that have been reported by other researchers, although not all of them had been placed in the embryonic context. In addition to xenobiotic metabolism genes [81], [82], [83] and cell-cycle genes [25], [27], [65], [84], we found down-regulation of genes involved in fin regeneration, recently identified as an important target of dioxin exposure [85], [86], as well as regulation of extracellular matrix genes, also a known dioxin target [86], [87]. The observed changes in immune system and steroid metabolism are consistent with dioxin's immunotoxic [2], [88] and endocrine-disruptive [20], [89] activities. Thus, while our experimental approach was based on whole embryo RNA, we were able to identify several altered biological processes that would appear to be tissue-specific. Nonetheless, it is likely that some tissue-specific transcriptional changes were diluted beyond recognition in our whole-embryo RNA extracts.

Interestingly, generalized stress-responsive transcriptional changes were not strongly evident until late in the time-course (although categories associated with xenobiotic responses were altered early, largely due to *cyp1a*). Indeed, our results suggest the hypothesis that dioxin-induced teratogenicity is less dependent on outright toxic effects such as the generation of oxidative stress, than on altered gene regulation. As detailed above, the prime candidates for driving teratogenesis are genes involved in embryonic morphogenesis processes, calcium and iron metabolism, and mitochondrial function.

Many proteins interact conditionally rather than at all times and in all contexts; e.g., AHR dimerization with ARNT only occurs after ligand binding. Furthermore, such interactions may represent either adaptive or toxic responses to a stressor. We identified differentially expressed links – interactions that appeared or disappeared upon dioxin exposure – and network clusters shaped by such interactions. Differentially expressed links were much less common than differentially expressed genes. This feature made the effects easier to localize and interpret, despite the greater complexity of networks as compared to gene sets. Most of the clusters were compact and relatively disjointed, which was expected since most of the transcriptome functioned normally even by the end of the experiment. We propose that “frontier” genes linked to both dioxin-enabled and dioxin-sensitive modules may be of particular importance in understanding dioxin's molecular effects. In future experiments, such segments may serve as “watch points” that would help distinguish adaptive from toxic responses.

In conclusion, we present the first published fish interactome, novel tools for interactome-based microarray data analysis, and intriguing microarray data that together suggest novel hypotheses regarding the mechanism of dioxin's toxicity. Future experiments will be required to verify the zebrafish-specific interactions that our interactome predicts, explore the relevance to other eukaryotic species as well as robustness and scalability of our bioinformatic tools, and test the biological hypotheses that our microarray data suggest, via morpholino or other techniques.

## Materials and Methods

### Dioxin exposures

AB* zebrafish embryos were exposed from 4 to 5 hpf to 1 ng/ml TCDD, a dose previously shown to induce deformities [37]. This dose would eventually cause mortality, but not until after the final sampling point (Henry et al., 1997). No dioxin-induced mortality was observed during the experiment. Dosing was done between 2 and 3 PM. Exposures were carried out at 28.5°C in a shaking incubator to maintain oxygen distribution. The carrier and control was 10 µl of DMSO per 10 mL 30% danieau in glass scintillation vials with Teflon-lined lids. TCDD was obtained from Cambridge Isotope (cat #ED-901-B, Solution Lot 35124-51, analyzed concentration 51.36 µg/ml). Initially, embryos were dosed in 3 vials with ∼80 embryos per vial. After 1 h, the dosing solution was removed and embryos in each vial were rinsed 3 times, transferred to a single clean vial (containing all ∼240 embryos) and rinsed 3 times more, and finally randomly distributed into into 12 clean vials in groups of approximately 15–20 embryos per vial with 5 mL danieau. Water was changed daily. 3 vials per treatment group were harvested daily on days 1 & 2; 2 vials per treatment were harvested on days 3, 4 and 5. The entire experiment was repeated 4 times independently (*n* = 4), approximately one week apart in time, except for day 2 under TCDD treatment where one sample degraded (*n* = 3) (Fig. 2A). The experimental protocol was approved by the Duke University Institutional Animal Care & Use Committee (IACUC).

### RNA extraction and microarray analysis

RNA from TCDD- and vehicle control-treated zebrafish was extracted daily between 10 and 11 AM for each time point. RNA extraction was carried out using Qiagen's RNEasy kit (Qiagen, Valencia, CA, USA), and analyzed for integrity with a BioAnalyzer (Agilent Technologies, Santa Clara, CA, USA). A universal reference design was used for this study, such that all control and TCDD samples were compared to pooled RNA extracted from a zebrafish embryonic fibroblast cell line. RNA was extracted as described above from Zf4 cells derived from 1-day old zebrafish embryos [90] grown in 25cm culture plates. Aliquots of the isolated RNA were stored in either liquid nitrogen or a −80C freezer until they were used for microarray experiments.

Microarray hybridizations were performed on the 22K Agilent platform. 1 µg of total RNA from each sample was amplified and labeled with a fluorescent dye (either Cy3 or Cy5) using the Low RNA Input Linear Amplification Labeling kit (Agilent Technologies, Palo Alto, CA) following the manufacturer's protocol. The amount and quality of the fluorescently labeled cRNA was assessed using a NanoDrop ND-1000 spectrophotometer and an Agilent Bioanalyzer. Equal amounts of Cy3- or Cy5-labeled cRNA were hybridized to the Agilent Zebrafish Oligo Microarray (Agilent Technologies, Inc., Palo Alto, CA) for 17 hrs, prior to washing and scanning. Data were extracted from scanned images using Agilent's Feature Extraction Software (Agilent Technologies, Inc., Palo Alto, CA). Microarray data are described in accordance with MIAME guidelines, have been deposited in the National Center for Biotechnology Information's GEO and are accessible through GEO series accession number GSE15410.

### Ortholog-supported creation of FunCoup network

An interaction network of zebrafish proteins was produced with data from other eukaryotes. Hence, only protein pairs with orthologs to at least one of those model species (MS) were included. There were 9377 such proteins, which produced 747333 links at our minimal confidence level of FBS>3.0 (Fig. S3). Confidence levels for FunCoup are calculated as the probability of functional coupling of two nodes, based on the Final Bayesian Score (FBS) associated with the strength of evidence for that interaction (described below). We employed Zfin IDs (ZDB-*) for both finding orthologs and presenting the whole predicted network in the web database. However, the web database allows querying for the other conventional IDs, such as gene symbols, ENSEMBL genes, proteins etc. Orthologs were identified using the InParanoid (version 5.0) database, and links were identified using FunCoup.

See in Methods S1:

1. identification of orthologs and ortholog-based network links,
2. identification of microarray-derived network links,
3. confidence estimates;
4. ANOVA of microarray changes.

### Generation of the time-dependent GO network

To generalize the network view and show the functional flow in the course of embryonic development affected by the experimental conditions, we constructed an (altered sub-) network of GO categories. It was based on the set of 898 dioxin-regulated (differentially expressed between the dioxin and control conditions; ANOVA, *p_α_*<0.01) genes assigned to a GO biological process and having a FunCoup link (confidence FBS>3).

1. For any two GO “biological process” categories, a link was counted if it connected differentially expressed (DE) genes – category members – in the original gene network.
2. The GO-GO links were classified into 9 *day patterns* according to the days when the genes were DE for the first time:
  - **day 1 -- day 1 : both genes DE on d.1;**
  - **day 1 → day 2 : one gene DE on d.1, the other on d.2;**
  - **day 2 -- day 2 : both genes DE on d.2;**
  - **day 2 → day 3 : one gene DE on d.2, the other on d.3;**
  - **day 3 -- day 3 : both genes DE on d.3;**
  - **day 3 → day 4 : one gene DE on d.3, the other on d.4;**
  - **day 4 -- day 4 : both genes DE on d.4;**
  - **day 4 → day 5 : one gene DE on d.4, the other on d.5;**
  - **day 5 -- day 5 : both genes DE on d.5;**
3. For each potential GO-GO network link, its enrichment was calculated as a chi-square score:
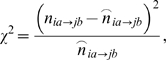

4. It compared the observed number 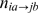 of links between GO categories *i* and *j* for the day pattern *a*→*b* with the expected one defined as 
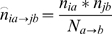
, i.e. calculated from occurrences of *any* links that involved genes of these categories expressed on days of interest *a* and *b*, respectively. *a* = {1, 2, 3, 4, 5} and *b* = {1, 2, 3, 4, 5}.
5. Only enriched GO-GO links were plotted in the GO network, i.e. ones with chi-square score >6.64 (*p_0_<0.01*), observed number of gene-gene links 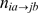
≥3, and the respective expected number 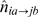
>0.5.

### Cytoscape analysis

#### Rosetta Resolver analysis

As described, a universal reference design was used for this study. The gene expression data were loaded into the Rosetta Resolver® Gene Expression Analysis System version 6.0.0.0.311. The data from biological replicate samples were combined using an error-weighted average and then “re-ratioed” in order to make direct comparisons between the TCDD treated samples and the vehicle control samples at each time point. These fold-change and p-value data were used for Cytoscape analyses.

#### Interactome

We used two different interactome versions for all comparisons in Cytoscape 2.6.0 [29], [40]. They were the FunCoup networks containing all interactions with a FBS>6 or FBS>8, in each case supplemented by a small number of literature-derived manually-curated interactions involved in the AHR pathway and the antioxidant responsive element pathway. These interactomes contained 5760 nodes and 99570 interactions (FBS>6) and 3512 nodes and 32520 interactions (FBS>8), and are available as .sif files in Data Files S3 and S4. The smaller interactome (FBS>8) consists of interactions for which there was more evidence and therefore obtained higher confidence. The larger interactome (FBS>6) includes, in addition, lower-confidence interactions but covers a greater number of the transcripts represented on the array. We chose to use both in our analyses, and then compare the results.

#### Identification of sub-networks with many differentially-expressed genes

We used the jActiveModules (version 2.2) plugin [29], [40] to identify the 20 most highly dioxin-altered network neighborhoods (modules) for each timepoint (days 1–5). We used an overlap threshold of 0.5 with size-adjusted scoring and regional scoring enabled. The greedy search was limited to a depth of 1 and a maximum depth from start nodes of 1. A separate search was initiated for each node in the network. These sub-networks can be retrieved by opening Cytoscape sessions (.cys files) containing the day-by-day jAM outputs (archived at http://funcoup.sbc.su.se/zfish_supplementary.html).

#### Identification of GO enrichment of active modules

We then used the BiNGO (version 2.0) plugin [91] to analyze the enriched Gene Ontologies for at least the top 13 modules for each day and interactome (5 days*2 interactomes = 10 total comparisons). We assessed overrepresentation using the hypergeometric test with the Benjamini and Hochberg false discovery rate (FDR) multiple testing correction and significance *p_FDR_*<0.05, testing each cluster versus the entire annotation and identifying altered GO Biological Processes. In some cases, BiNGO identified biological processes within individual active modules that consisted almost entirely of genes that either were not present on the microarray, or did not show dioxin alteration either by fold-change or by p-value. Such processes were identified via manual inspection and eliminated from consideration.

### GoMiner analysis

The ratioed gene expression data were used to identify differentially expressed transcripts using the following thresholds: log ratio p-value<0.1, absolute fold-change>1.3, log_10_ intensity>2.5, in at least 3 of 4 replicates on each day. Gene ontology enrichment analysis was performed using High-Throughput GoMiner [38], [39] on the differentially expressed transcript list that was generated for each time point; separate analyses were performed for lists of up-regulate, down-regulated, and changed (i.e. both up- and down-regulated) genes.

## Supporting Information

Methods S1 This file contains supplemental methods.
(0.13 MB DOC)

Figure S1 Heat map of genes significantly changed in the course of the 5-day transcriptome observation after dioxin treatment.
(0.31 MB TIF)

Figure S2 Distribution of fold-change values in microarray probes significantly (pα<0.01) differentially expressed between dioxin-treated and control zebrafish embryos.
(0.02 MB TIF)

Figure S3 The overlap between a FunCoup network based chiefly on data from orthologs and a network with links based on general mRNA co-expression in the zebrafish microarray dataset over 39 experimental conditions.
(0.06 MB TIF)

Figure S4 Evidence used for the generation of the FunCoup network came from 8 eukaryotic species (top panel A) and 51 individual large-scale datasets that belong to 8 major data types (bottom panel B).
(0.05 MB TIF)

Figure S5 Connectivity distributions of genes in the zebrafish interactome. Distribution of connectivity k (number of links per network node) in biological networks is scale-free, i.e., characterized by high occurrence P(k) of nodes (genes) with few links, while links with multiple connections, so called network hubs, are rare. This is modeled with a power law. When plotted on log-log scale, points produce a straight line. A. Orthology-based network at the lowest confidence cutoff FBS = 3; B. Orthology-based network at a stricter confidence cutoff FBS = 7; C. Microarray-based links with orthology support (FBS>3) enabled with dioxin treatment (“E”); D. Microarray-based links with orthology support (FBS>3) sensitive to (disappearing after) dioxin treatment (“S”).
(0.08 MB TIF)

Figure S6 Combined perspective on the network perturbation after the dioxin treatment during the five days of the experiment. Nodes of the network are defined as GO “biological process” categories that include one or more differentially expressed genes in the course of the experiment. Network edges summarize those in the gene network that connect differentially expressed genes between GO-GO. Node color represents the fraction of the genes in that node that are regulated on any day (green is low, red is high). Edge thickness and opacity represent the number of gene-gene links between two categories and chi-square score that this pair of categories is enriched in links, respectively. Edge color and arrows show timing of differential expression between gene-gene pairs in respective GO categories (see legend). A user-manipulated Cytoscape map with break-down to individual days is presented as Data File S8.
(1.61 MB TIF)

Figure S7 The most-altered neighborhood (by jActiveModules in Cytoscape) identified on day 1 post-dioxin treatment is characterized by multiple genes that exhibit statistically significant but small fold changes. Red node color indicates upregulation and green indicates downregulation, with the faintest coloration indicating a 1.3-fold change and darker coloration indicating greater change. Node border color indicates significance; the faintest blue indicates pα = 0.05, with darker blue indicating greater statistical significance (by Rosetta Resolver).
(1.73 MB TIF)

Figure S8 Many genes in an altered neighborhood (by jActiveModules in Cytoscape) identified on day 3 are involved in stress response signaling and extracellular matrix. Red node color indicates upregulation and green indicates downregulation, with the faintest coloration indicating a 1.3-fold change and darker coloration indicating greater change. Node border color indicates significance; the faintest blue indicates pα = 0.05, with darker blue indicating greater statistical significance (by Rosetta Resolver).
(0.08 MB TIF)

Data File S1 Correlation (across all transcripts) for each microarray.
(0.05 MB XLS)

Data File S2 A list of altered genes identified as dioxin-modulated by ANOVA analysis, along with the days on which they were differentially expressed (DE) and basic descriptions and GO annotations.
(0.51 MB XLS)

Data File S3 Zebrafish interactome (FBS>6) in .sif format.
(0.47 MB ZIP)

Data File S4 Zebrafish Interactome (FBS>8) in .sif format.
(0.15 MB ZIP)

Data File S5 GO enrichment analysis of modules enriched in dioxin-enabled (P), -sensitive (N), or a combination (PN).
(0.06 MB XLS)

Data File S6 Genes that were dioxin-regulated on day 1 (“day 1”), ordered by the number of genes that were dioxin-regulated on days 2–5 to which each “day 1” gene was linked in the interactome.
(0.02 MB XLS)

Data File S7 Genes that were dioxin-regulated on day 1 (“day 1”), listed on the same row with each gene that was dioxin-regulated on days 2–5 (“Other day”) and to which the “day 1” gene was linked in the interactome.
(0.32 MB XLS)

Data File S8 GO-GO Cytoscape session file.
(0.18 MB ZIP)

Data File S9 GO enrichment analysis of all differentially expressed genes (by ANOVA) for all possible contrasts (treatment and day combinations; see Methods).
(0.44 MB XLS)

Data File S10 GO enrichment analysis of all dioxin-modulated genes (by ANOVA), grouped by day.
(0.04 MB XLS)
