# Supplementary material for: Dynamic Zebrafish Interactome Reveals Transcriptional Mechanisms of Dioxin Toxicity
Source: PLoS One. 2010 May 5;5(5):e10465. doi: 10.1371/journal.pone.0010465 (PMC2864754; doi:10.1371/journal.pone.0010465)
Supplement: Methods S1 — This file contains supplemental methods. (0.13 MB DOC) [file pone.0010465.s001.doc]

Supplementary Methods

**Dynamic Zebrafish Interactome Reveals Transcriptional Mechanisms of Dioxin Toxicity**

Andrey Alexeyenko^1^, Deena M Wassenberg^2^, Edward K Lobenhofer^3^, Jerry Yen^2^, Erik LL Sonnhammer^1^, Elwood Linney^2^, Joel N Meyer*^4^

### Overview of Interactome generation

Normally, FunCoup [1] combines a species’ own evidence with that from orthologs. However, a network in a species without any of its own data can also be generated. FunCoup can predict gene-gene interactions by simply correlating gene expression profiles over a sufficiently extensive series of conditions. Our expression dataset was the only source of *D. rerio* data suitable for this purpose (i.e. relevant to protein function discovery and large enough to calculate correlations with confidence). We thus decided to use it as part of the evidence for building the backbone zebrafish interactome with FunCoup. Importantly, co-expression evidence from the zebrafish microarray data taken alone was not strong enough to define a single link. The interactome connections between pairs of genes, predicted from data integration in FunCoup, received continuous Final Bayesian scores (FBS) – probabilistic estimates of links’ confidence. In the course of training on a large set of known interactions (signaling and metabolic zebrafish pathways available in KEGG database), each particular value of each metric pair-wise similarity (correlations etc.) could have been quantified as evidence of functional coupling between the two genes, depending on which dataset and which organism it comes from. Thus the FBS scores summarized the evidence for a link between the genes from a range of eukaryotes plus a correlation over our zebrafish microarray dataset. The maximum FBS from zebrafish co-expression evidence was 3.23, and thus interactions almost never (<0.01%) surpassed our minimum threshold of FBS=3 (*p_FDR_* < 0.50; calculation of FBS and estimation of false discovery rate described below) based on the microarray data alone. Thus, the FunCoup network could not have been created without data from other organisms, and was in fact comprised almost entirely of genes with orthologs in at least one better studied eukaryote. At FBS > 3, 9,377 such genes produced a total of 747,333 interactions in the FunCoup network (Fig. S3). Nonetheless, a large fraction of FunCoup interactions (27.3%) would not have reached FBS=3 without the help of the zebrafish microarray correlations. Underscoring the importance of this data, its overall contribution to the construction of the network (i.e. an average contribution to confidence of a single link) was ~17.3%, (Fig. S4A). In summary, the zebrafish microarray correlations played an important but largely supportive role in the generation of the FunCoup network, such that its use did not compromise our ability to retrieve dioxin-responsive information from the same data. The details of the latter are described in the next section.

Importantly, it is possible to restrict the interactome to higher-confidence interactions. For example, at confidence FBS>7, the network comprised 52,139 interactions between 4,313 genes (at threshold *p_FDR_* = 0.15: least confident interactions had an FDR of 0.15; most had much lower *local* FDRs). For the jActiveModules analyses, we used cutoffs FBS>6 and FBS>8 (92425 and 30449 links, respectively; see Methods for more details). No matter what interactome is chosen, the potential for false interactions to mislead subsequent analysis is minimized by searching for regions of the interactome that are enriched in *many* altered genes or interactions. This is the case for the approaches we took (jActive modules, CohTop, GO-GO).

To verify that the interactions based on information from orthologs were still relevant in the zebrafish interactome context, we analyzed the agreement of the correlation-derived zebrafish microarray-based links (MABL) with those based on other species’ data. Of note, in the data integration framework of FunCoup, high co-expression in any set is not required to reach a higher FBS score. For example, of 50,369 FunCoup links with experimentally known physical interaction in orthologous proteins, roughly 70% did not have significant support from co-expression (either zebrafish data set or orthologs). This is not surprising; for example, protein complex members are co-regulated in various ways, not necessarily by transcription [2].

Nonetheless, we found that 21.7% of the FunCoup links were also co-expressed in *D. rerio* as defined by MABL (4,902 out of 22541 links with FBS *from other species* >7 and available zebrafish microarray profiles, while the general occurrence of co-expression at this level among zebrafish gene pairs with microarray data orthologs was only 4.2%). Thus, despite the methodological discrepancies between our interaction datasets and the large evolutionary distances from *D. rerio* to other eukaryotic organisms employed, the FunCoup link set based on non-fish evidence had high overlap with the MABL (Fig. S3). This supports the validity of our FunCoup/InParanoid-based approach to interactome creation.

Gene interaction networks are often characterized as scale-free, i.e. manifest a power-law distribution of connectivity (number of interactions per gene). On the contrary, networks generated randomly do not contain any biological information, and are expected to show a normal distribution of node connectivity. We determined that our networks are perfectly scale-free, both at FBS>3 and FBS>7. The same feature was observed in the interactions sensitive to and enabled by dioxin derived from the microarray data, supporting the biological basis of our interactome (Fig. S5).

As an independent validation, we considered how well FunCoup recapitulated two pathways central to the present work – the combined aryl hydrocarbon receptor and hypoxia pathways, and the antioxidant response element network. Both were compiled manually by us via expert knowledge and literature search. The FunCoup network, at the lowest confidence level and without using any zebrafish data, contained 50 links between 24 members of the former and 6 links between 8 members of the latter (<http://funcoup.sbc.su.se/zfish_supplementary.html>). The genes – pathway members –also had numerous novel links to other genes in the network, and we used those in our analysis.

### Ortholog identification

Orthologs were obtained from the InParanoid 5.0 database [3,4]. The InParanoid software finds multiple members of ortholog clusters as a result of post-speciation gene duplications, a particularly critical consideration in zebrafish [5]. In the seven eukaryotic species that provided information, 19.4-58.7% of zebrafish orthologs had many-to-one or many-to-many relations (in 8.6-32.0% of distinct ortholog clusters). In such clusters, distinguishing functionally optimal counterparts (functional orthologs) from sequence similarity alone was not possible. We had shown [1]that to transfer information across species, alternative ortholog pairs should be treated equally, and functionally best values (e.g. highest correlation of expression profiles) be selected to characterize potential links. Thus, for a potential link between zebrafish genes *A* and *B*, their (often) multiple co-orthologs (*m* orthologs of *A* and *n* of *B*, respectively) should have been tested for positive pairwise metrics in any of *m*×*n* pairs. This procedure was repeated for each of the species where both *A* and *B* had orthologs.

### Identification and evaluation of network links

We recently created FunCoup, a public database of gene interaction networks [1]. This deeply optimized technology integrates multiple datasets containing interaction information into a single network (interactome), and quantifies the strength of the evidence for each integration. We used interaction information from 51 individual, large-scale datasets from 7 eukaryotic species (apart from the zebrafish expression set) (Fig. 2). Datasets included physical protein-protein interactions (both high-throughput and verified in small-scale experiments), sub-cellular co-localization, results of transcription factor (TF) and miRNA targeting discovery, similarity of phylogenetic profiles (co-presence of orthologs in a series of genomes), and similarity of mRNA and protein expression profiles.

In each set of inparalog pairs with data, functionally best (e.g. the highest Pearson linear correlation coefficient, or PLC) values of the metrics were evaluated in the Bayesian network that obtains likelihood values by training on “gold standards” of functional coupling (FC), i.e. sets of known functionally coupled protein pairs. Using the KEGG pathway database [6], we compiled a set of functional links such that: 1) the two proteins were members of the same KEGG pathway (metabolic or signaling) and 2) either the pathway was compact (< 30 and < 20 members, respectively) or this same pair was also found in another pathway. After training, the FunCoup predictor learned that e.g. gene pairs co-expressed as PLC >0.932 were enriched in members of the training set ~25-fold, and thus received a log (base *e*) likelihood score of functional coupling 3.22. Conversely, gene pairs with practical absence of co-expression, i.e. PLC in the range [-0.633…0.538), were 1.15-fold depleted in functionally coupled examples – hence log likelihood –0.14. The whole range of PLC was discretized into 10 bins, and each received a distinct log likelihood score, either positive or negative. Data from orthologs in model organisms were used in the same way (processed with special similarity scores for co-localization in a cellular compartment, physical interactions etc. – see Alexeyenko and Sonnhammer 2009 for more details). Then using available data, all possible gene-gene pairs in the genome were evaluated, while the sum of log likelihood values called *Final Bayesian Score* (FBS) indicated confidence of functional coupling for each pair. When FBS exceeded a predefined cutoff (we chose FBS=3 as it usually corresponds to ~1% of all possible gene-gene links) the pair of genes was deemed functionally coupled. The two genes were then connected with an edge in the network (see details in *Materials and Methods*). This measure of confidence was based on the availability and amount of evidence integrated for a particular interaction, rather than quantifying the inherent strength of that interaction. Therefore, it was possible that lower-confidence links represent interactions lacking evidence for technical reasons (genes not included on microarrays, false negatives in yeast two-hybrid screens due to inappropriate biological context, etc.). For this reason, upon selection of a suitable confidence cutoff, we equally treated all links in the produced network.

To investigate the credibility of the predicted links, we applied random shuffling of additive pieces of evidence, and determined that the global *false discovery rate* (FDR, [7] at FBS>3 did not exceed 50%.

### Microarray-based links

We identified interactions based on statistically significant co-expression of pairs of genes in our microarray dataset. This analysis resulted in identification and labeling of links that existed only in the presence of dioxin, only in the absence of dioxin, regardless of dioxin exposure, and through development. Statistically significant correlation of expression was analyzed by ANOVA, controlling for the false discovery rate (FDR), as described below.

### *Analysis of variance: definition of altered genes and pairs of genes*

Generally, the analysis of variance (ANOVA;[8]) conveys a probability that an effect does *NOT* influence the outcome (gene expression in our case). The result of this probabilistic test depends on 1) the effect strength – the variability due to the effect of interest, 2) the unexplained, or *residual*, variability (experimental and measurement errors, unaccounted factors etc.), and 3) sample size. The probability is assigned as a function of the variability arguments (sums of squared differences *SS* between the mean and the random variable realization) and the sample size arguments – degrees of freedom (*df*). The effect is declared significant if the ratio of the “effect A” variability to the residual variability

$$F_{A}=\frac{mS_{A}}{mS_{e}}$$

is higher than a pre-defined standard level *F_st_*. The mean squares *mS* are sums of squares *SS* divided with respective *df*, i.e. $mS=\frac{SS}{df}$ .

For example, we considered the effect DAY significant at *p_0_*<0.01 when:

>7.60,

because the probability of the null hypothesis *H_0_:” the factor DAY has NO effect*” at the standard value *F_α=0.01;1,29_=*7.60 was 0.01 for *df_numerator_*=1 and *df_denominator_*=29.

In this framework, we were able to analyze the response of both individual genes and *pairs* of genes, as putative edges in the interactome. In the former case (dubbed **2W**), we performed a 2-way ANOVA with factors DAY, TREATMENT, and their interaction DAYxTREATMENT (Fig. 1A in the main text). In the latter case (**3W**), there was a 3-way ANOVA – in addition to the two factors, we analyzed GENE, and interactions TREATMENTxGENE, DAYxGENE, and DAYxTREATMENTxGENE. For each analyzed entry – an individual gene in **2W** and a putative gene link in **3W** – we determined *p*-values (*p_α_*, i.e. probability of a non-perturbed entry to be erroneously claimed perturbed) for main factors’ effects, and effects of their interactions. The main effect GENE in **3W** only informed on the difference in the average mRNA concentration between the two genes. Therefore, it was not of practical interest: a pair of genes might operate in a coupled mode even if their absolute abundance differed by several orders of magnitude.

Hence, to discover for example genes that changed expression in the time course of the experiment (i.e. effect DAY significant), we looked at items with sufficiently high *F_DAY_* (>7.60 which corresponded to *p_α_*<0.01).

The *contrasts*, or planned comparisons, are ANOVA models establishing significant differences between *sets of conditions (levels)* of a factor rather than this factor’s significance in general.

For example, when we were specifically interested in expression change between days {1,2} (set *X*) and days {3,4,5} (set *Y*), then the contrast was defined with:

,

where $\bar{e}_{Xi}$ and are the group means of day *i=*{1,2} and day *j=*{3,4,5}, respectively. *m* is the average group size, i.e. number of observations on each day. The coefficients *κ_X_* and *κ_Y_* were set such that *n_X_κ_X_* = *n_Y_κ_Y_*.

The significance of the *contrast XY* was proven if the ratio $\frac{mS_{contrastXY}}{mS_{e}}$was higher than the standard threshold *F* for the respective numbers of degrees of freedom. For the contrasts with reduced number of observations (e.g. at dioxin=NO), the number of summation items and *df* were respectively changed.

Identically to the main factor and interaction analysis in **2W** and **3W** described above, the contrast analysis was applied to both individual genes and their pairs.

### *Confidence (False Discovery Rate)*

$E(FDR)\leq\frac{FP}{N_{total}}\alpha\leq\alpha$

In the ordered list of p-values for a series of individual (differential expression) tests, we compare each next (ascending from highest, i.e. worst to lowest, i.e. best) value *p_(i)_* to $\frac{i}{N_{total}}\alpha$. The first *i* value at which the condition is satisfied sets the false discovery rate below $\frac{i}{N_{total}}\alpha$ for the top *i* tests (Benjamini and Hochberg 1995). Respectively, for a fixed *α* we obtain:

$p_{FDR}\leq\frac{i}{N_{total}}\alpha$for all tests with *p≤α*. Although, as the p-values quickly decreasing in the list, the overall fraction of false discoveries is much lower.

### *Zebrafish microarray-derived condition-specific links*

When applied to a pair of gene profiles, the experimental design allowed measuring effects of factors “dioxin treatment”, “developmental stage”, and “gene” as well as any of their combinations. The procedure could be executed under the terms of the standard 3-way factorial ANOVA.

We performed the correlation analysis (Pearson linear correlation coefficient, PLC) and ANOVA on each of the 281,188,755 pairs possible between the 23,715 distinct gene/probe IDs. Securing the effects’ confidence at a formally acceptable level *p*_α_ < 0.05, we focused on employing the variability terms to select network links with desirable functional properties. In the *Confidence* section above we evaluate the respective statistical significance.

Thus, each link in the resulting network was produced by expression analysis of 2 genes in the course of embryonic development and characterized by labels indicating at least one of the following:

1. Strong correlation *independent* of the dioxin treatment, i.e “dioxin-resistant” link (label “R”); strong correlation observed *either* only after the dioxin treatment *or* only in the absence of it (dioxin-enabled and dioxin-sensitive, labels “E” and “S”, respectively);
2. Strong correlation with a significant developmental pattern (label “D”), must be synchronous between the two genes.

Labels “E” and “S” in (a) were assigned given the following conditions hold:

1. $\text{max(|PLC}_{D+}\text{|, |PLC}_{\text{D-}}\text{|)}>\text{c}_{\text{minPLC}}$;

i.e. at least one PLC value exceeds the threshold 0.85 (see below). PLC_D+_ referes to the PLC value for the dioxin-treated samples, and PLC_D-_ refers to the PLC for the control samples.

1. $\frac{\text{|PLC}_{D+}\text{- PLC}_{\text{D-}}\text{|}}{\text{max(|PLC}_{D+}\text{|, |PLC}_{\text{D-}}\text{|)}}>c_{diffPLC}$;

i.e. the difference between PLC_D+ and_ PLC_D-_ is strong enough.

1. $F_{TREAT}>F_{st}(p_{\alpha}=0.05,df_{1}=1,df_{2}=19)$ ***OR*** $F_{TREATxGENE}>F_{st}(p_{\alpha}=0.05,df_{1}=1,df_{2}=19)$ ***OR*** $F_{DAYxTREATxGENE}>F_{st}(p_{\alpha}=0.05,df_{1}=1,df_{2}=19)$,

i.e. at least one of the three effects (F-ratios) must be significant at *p_0_<*0.05.

If ***neither*** “S” ***nor*** “E” was aassigned, the link was declared resistant (“R”), given $\text{PLC}_{full}>\text{c}_{\text{fullPLC}}$ ***and*** *F_TREAT_* < *F _α=0.05;1,19_*,.

Label “D” in (b) was assigned given ***all*** the three conditions hold:

1. *F_DAY_*>*F _α=0.001;4,19_*;
2. *F_DAYxGENE_*<*F _α=0.20;4,19_*;
3. $\text{PLC}_{full}>\text{c}_{\text{fullPLC}}$.

Cutoff values:

*c_minPLC_* = 0.85;

*c_diffPLC_* = 0.60;

*c_fullPLC_* = 0.85;

We calculated the false discovery rate of the dioxin-enabled and dioxin-sensitive links in the orthology-based network as follows:

Appearance of a FunCoup network link as co-expressed in either “dioxin” ***or*** “control” condition and NOT co-expressed in the other one (exclusive OR) was modeled as co-incidence of 3 independent events. The two alternative events (manifested in either “dioxin” or “control” condition) are statistically indistinguishable and treated here identically. Errors of Type I, i.e. a falsely observed correlation when there was none in the reality, had the estimated error rate:

*p_α_*(PLC_D+_ > *c_min_*_PLC_) = *p_α_*(PLC_D–_ > *c_min_*_PLC_) = 0.006.

As the complementary probabilities (1 - *p_α_*) are close to 1, then the combined probability of the conditions (1) ***and*** (2) is:

*p_α_*(PLC_D+_ > *c_min_*_PLC_; PLC_D–_ < *c_min_*_PLC_) + *p_α_*(PLC_D+_ < *c_min_*_PLC_; PLC_D–_ > *c_min_*_PLC_) – *p_α_*(PLC_D+_ > *c_min_*_PLC_; PLC_D–_ > *c_min_*_PLC_) ≈ 0.011;

*p_α_* of condition (3), i.e. that ANOVA produced a falsely high F-ratio of dioxin/control difference or its interaction, was relaxed due to the OR condition:

1 – (1 – *p_α_*(*F_TREAT_* > *F_st_*)) · (1 – *p_α_*(*F_TREATxGENE_* > *F_st_*)) · (1 – *p_α_*(*F_DAYxTREATxGENE_* > *F_st_*)) ) =

1 – 0.95^3^ = 0.143;

Thus, the estimate of *α*-error rate was:

*p_α_*(label “E” ***or*** “S” assigned) = 0.143 · 0.011 = 0.0016.

From the procedure of evidence random shuffling in FunCoup links, we established

*p_α_*(FBS>3) = 0.0075;

Then for the pairs between genes having both orthologous evidence for FunCoup and a zebrafish microarray profile (N_FunCoup&microarray_ = 6573), the joint probability is:

*p_α_*(FBS>3; label “E” ***or*** “S” assigned) = 0.0075 · 0.0016=1.2·10^-5^,

i.e. we expected the number of false FunCoup links with FBS>3 and a label “E” ***or*** “S” to be:

*p_α_*(FBS>3; label “E” ***or*** “S” assigned) · (N_FunCoup& microarray_ · (N_FunCoup&microarray_ – 1) / 2) =

1.2·10^-5^ · 21,598,878 = 260.3 links;

whereas the real number was 2338 links, and thus:

*p_FDR_*(FBS>3; label “E” ***or*** “S” assigned) = 260.3 / 2338 = 0.111.

### Analysis of a changing interactome with CohTop, a network clustering algorithm

Interactomes are dynamic. For example, some proteins interact with each other at a given developmental stage only, or only upon a given stimulus. We created an algorithm designed to detect modules of *top*ological *coh*esion CohTop. In principle, such an approach can be applied to any network. However, we analyzed only the subnetwork comprising links present in the FunCoup ***AND*** (***EITHER*** only upon exposure to dioxin – dioxin-enabled, “E”, ***OR*** only in its absence – dioxin-sensitive, “S”).

The algorithm of CohTop aims at finding such sub-network structures (clusters) that:

- 1. there is an uninterrupted path between any two given nodes – cluster members;
  2. the number of inward edges is maximized;
  3. the number of outward edges is minimized.

To find a reasonable trade-off between (2) and (3), at each step of the agglomerative procedure, each pair of distinct potential clusters {*i*,*j*} was evaluated for a potential “merge gain”, defined via the Kullback-Leibler divergence [9]. To measure the efficiency of network graph clustering,

To measure the efficiency of network graph clustering, we adapted the divergence as:

$nD_{KL}=\frac{N_{edges,total}}{N_{nodes}^{2}}\log\frac{\frac{N_{edges,total}}{N_{nodes}^{2}}+\alpha}{\frac{N_{edges,cluster}}{N_{nodes}^{2}}+\alpha}$,

where $N_{edges,total}=\sum_{x=1}^{N_{nodes}} n_{x}$and α is a small pseudo-count (α =0.1).

(*n_x_* being the total number of edges of the node *x*), and

$N_{edges,cluster}=\sum_{x=1}^{N_{nodes}} \sum_{y=1}^{N_{nodes}} (n_{d(x,y)}+\sqrt{n_{s(x,y)}})$ ,

i.e. to evaluate the internal connectivity of the cluster, all pairs {*x*,*y*} of *N_nodes_* members of the evaluated cluster were checked. $n_{d(x,y)}$ equaled 1 if *x* and *y* were linked ***directly*** in the network, and 0 otherwise. The number of ***shared*** network neighbors $n_{s(x,y)}$served as an indirect measure of mutual relevance of *x* and *y*, and was especially useful at earlier steps of the algorithm. The shared neighbors might not belong to the eventually formed cluster, but could point in advance at prospective clustering directions.

*nD_KL_* was only calculated for clusters that had at least 1 direct link connecting them.

The merge gain $MG=nD_{KL(i)}+nD_{KL(j)}-nD_{KL(i\cup j)}$ must be positive for the pair {*i*,*j*} to be considered further, i.e. the union of *i* and *j* must account for the network modularity better than *i* and *j* taken separated.

The cluster pairs were ranked by *MG*, and the *t* top pairs (*t* set to 100) were merged at each iteration step *I*. Each consecutive merge *m*={1…*t*} was allowed only if none of the two clusters had been merged earlier in a merge *m’<m*. Otherwise, the pair was skipped. Therefore, the number of practically performed merges at *I* was much lower than *t.*

In this form, the agglomeration was relatively well balanced, i.e. did not favour any particular size configurations, such as single genes added to largest clusters, or merging equally sized clusters. The procedure terminated when no further positive merge gain was expected among any pair of clusters. Depending on the network size and sparseness, it happened at *I*=15…50.

## References for Supplementary Methods

1. Alexeyenko A, Sonnhammer EL (2009) Global networks of functional coupling in eukaryotes from comprehensive data integration. Genome Res 19: 1107-1116.

2. de Lichtenberg U, Jensen LJ, Brunak S, Bork P (2005) Dynamic complex formation during the yeast cell cycle. Science 307: 724-727.

3. O'Brien KP, Remm M, Sonnhammer EL (2005) Inparanoid: a comprehensive database of eukaryotic orthologs. Nucleic Acids Res 33: D476-480.

4. Remm M, Storm CE, Sonnhammer EL (2001) Automatic clustering of orthologs and in-paralogs from pairwise species comparisons. J Mol Biol 314: 1041-1052.

5. Meyer A, Van de Peer Y (2005) From 2R to 3R: evidence for a fish-specific genome duplication (FSGD). Bioessays 27: 937-945.

6. Kanehisa M, Goto S, Kawashima S, Nakaya A (2002) The KEGG databases at GenomeNet. Nucleic Acids Res 30: 42-46.

7. Benjamini Y, Hochberg Y (1995) Controlling the false discovery rate: A practical and powerful approach to multiple testing. J R Stat Soc B 57: 289–300.

8. Huitson A (1966) The analysis of variance: Charles Griffin and company, Ltd.

9. Kullback S, Leibler RA (1951) On information and sufficiency. Ann Math Stat 22: 79-86.
