# Supplementary figures and images for: Dynamic Zebrafish Interactome Reveals Transcriptional Mechanisms of Dioxin Toxicity
Source: PLoS One. 2010 May 5;5(5):e10465. doi: 10.1371/journal.pone.0010465 (PMC2864754; doi:10.1371/journal.pone.0010465)

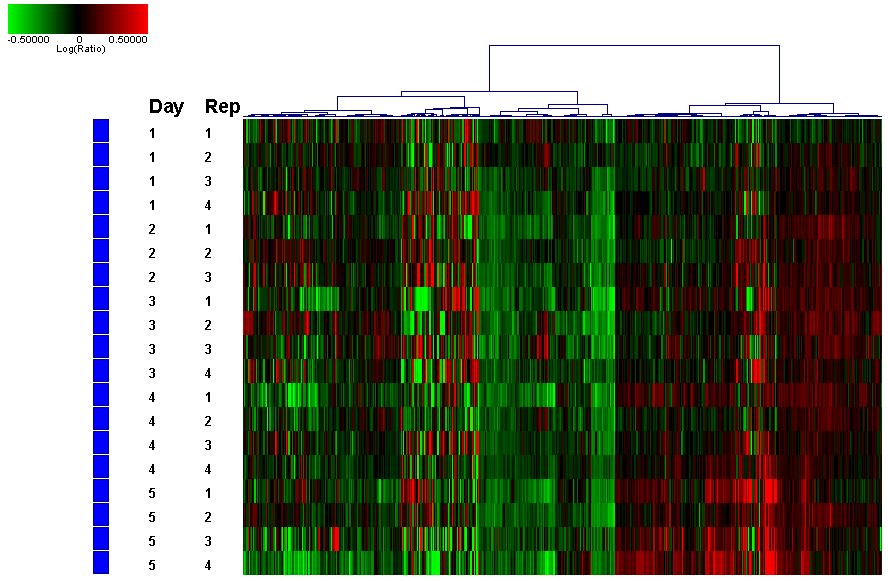

Supplement: Figure S1 — Heat map of genes significantly changed in the course of the 5-day transcriptome observation after dioxin treatment. (0.31 MB TIF) [file pone.0010465.s002.tif]

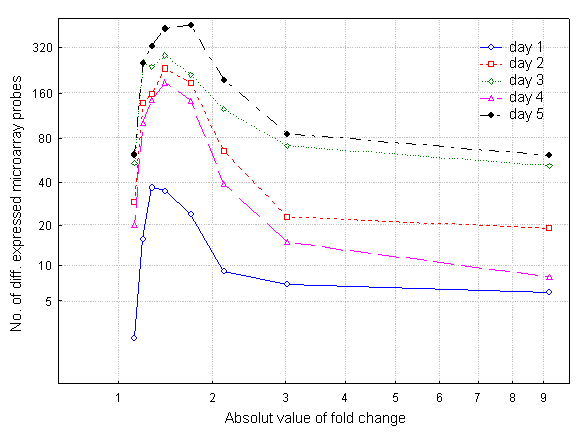

Supplement: Figure S2 — Distribution of fold-change values in microarray probes significantly (pα<0.01) differentially expressed between dioxin-treated and control zebrafish embryos. (0.02 MB TIF) [file pone.0010465.s003.tif]

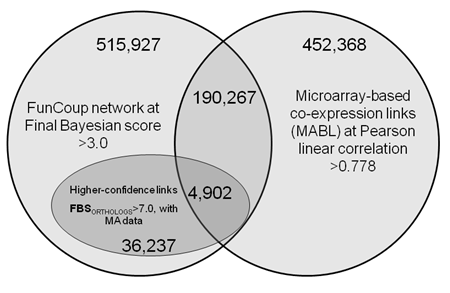

Supplement: Figure S3 — The overlap between a FunCoup network based chiefly on data from orthologs and a network with links based on general mRNA co-expression in the zebrafish microarray dataset over 39 experimental conditions. (0.06 MB TIF) [file pone.0010465.s004.tif]

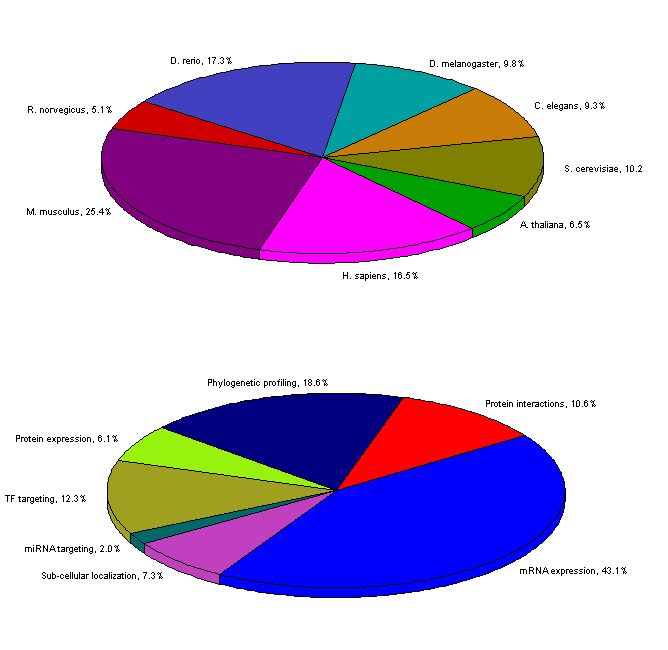

Supplement: Figure S4 — Evidence used for the generation of the FunCoup network came from 8 eukaryotic species (top panel A) and 51 individual large-scale datasets that belong to 8 major data types (bottom panel B). (0.05 MB TIF) [file pone.0010465.s005.tif]

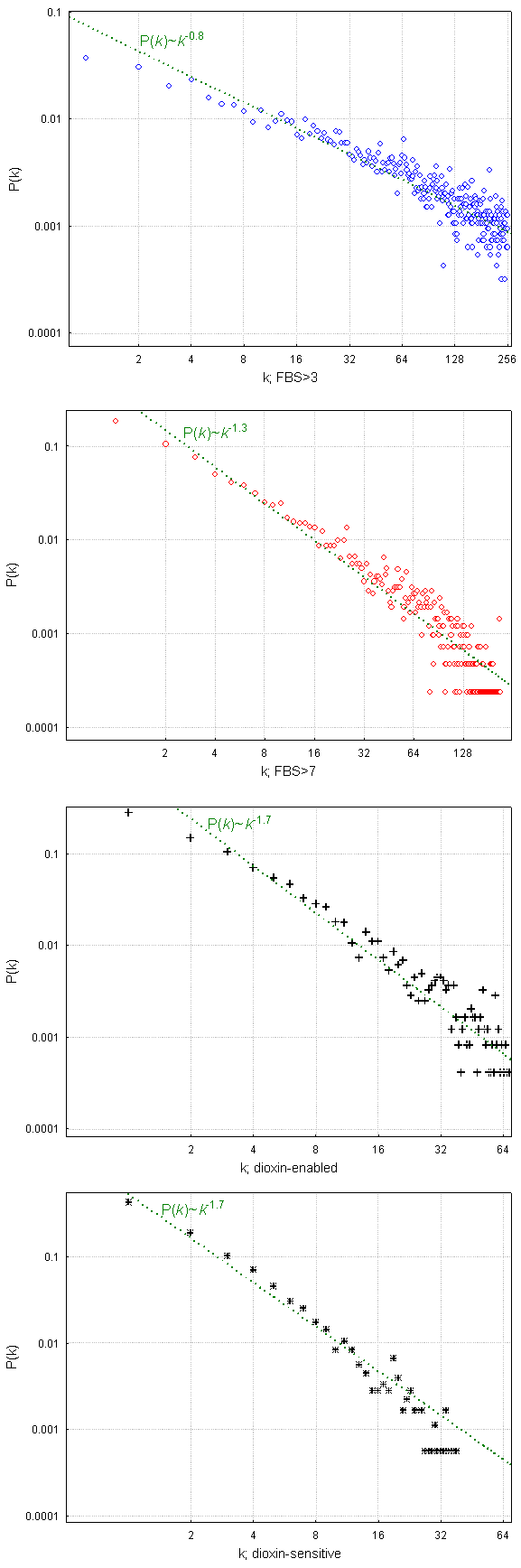

Supplement: Figure S5 — Connectivity distributions of genes in the zebrafish interactome. Distribution of connectivity k (number of links per network node) in biological networks is scale-free, i.e., characterized by high occurrence P(k) of nodes (genes) with few links, while links with multiple connections, so called network hubs, are rare. This is modeled with a power law. When plotted on log-log scale, points produce a straight line. A. Orthology-based network at the lowest confidence cutoff FBS = 3; B. Orthology-based network at a stricter confidence cutoff FBS = 7; C. Microarray-based links with orthology support (FBS>3) enabled with dioxin treatment (“E”); D. Microarray-based links with orthology support (FBS>3) sensitive to (disappearing after) dioxin treatment (“S”). (0.08 MB TIF) [file pone.0010465.s006.tif]

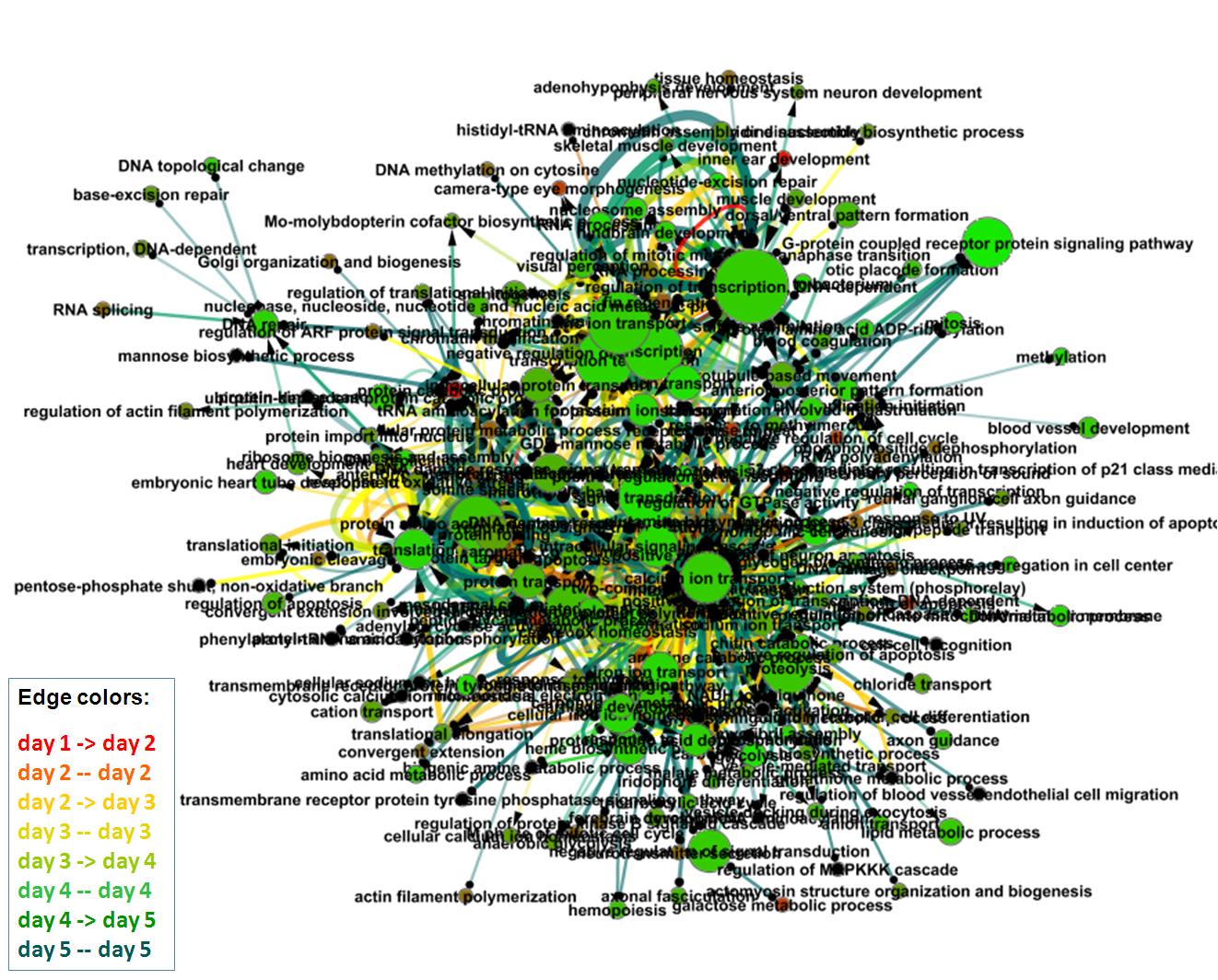

Supplement: Figure S6 — Combined perspective on the network perturbation after the dioxin treatment during the five days of the experiment. Nodes of the network are defined as GO “biological process” categories that include one or more differentially expressed genes in the course of the experiment. Network edges summarize those in the gene network that connect differentially expressed genes between GO-GO. Node color represents the fraction of the genes in that node that are regulated on any day (green is low, red is high). Edge thickness and opacity represent the number of gene-gene links between two categories and chi-square score that this pair of categories is enriched in links, respectively. Edge color and arrows show timing of differential expression between gene-gene pairs in respective GO categories (see legend). A user-manipulated Cytoscape map with break-down to individual days is presented as Data File S8. (1.61 MB TIF) [file pone.0010465.s007.tif]

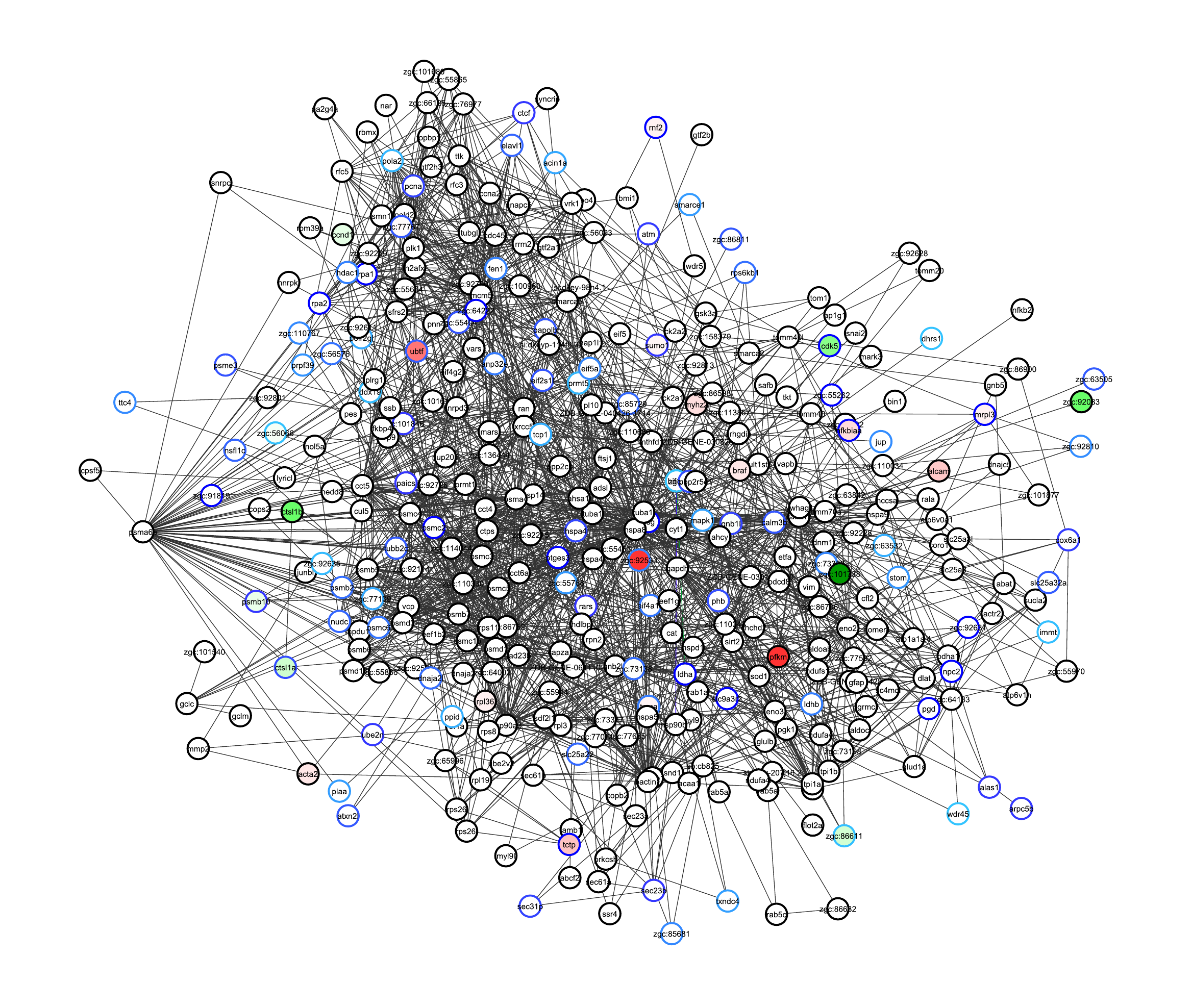

Supplement: Figure S7 — The most-altered neighborhood (by jActiveModules in Cytoscape) identified on day 1 post-dioxin treatment is characterized by multiple genes that exhibit statistically significant but small fold changes. Red node color indicates upregulation and green indicates downregulation, with the faintest coloration indicating a 1.3-fold change and darker coloration indicating greater change. Node border color indicates significance; the faintest blue indicates pα = 0.05, with darker blue indicating greater statistical significance (by Rosetta Resolver). (1.73 MB TIF) [file pone.0010465.s008.tif]

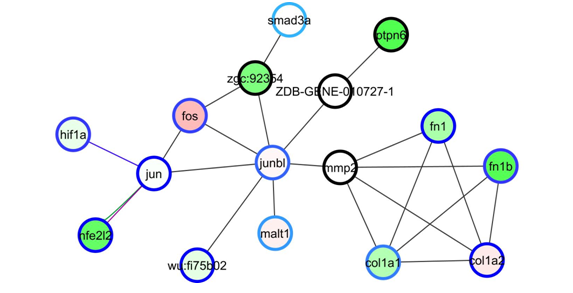

Supplement: Figure S8 — Many genes in an altered neighborhood (by jActiveModules in Cytoscape) identified on day 3 are involved in stress response signaling and extracellular matrix. Red node color indicates upregulation and green indicates downregulation, with the faintest coloration indicating a 1.3-fold change and darker coloration indicating greater change. Node border color indicates significance; the faintest blue indicates pα = 0.05, with darker blue indicating greater statistical significance (by Rosetta Resolver). (0.08 MB TIF) [file pone.0010465.s009.tif]
